# A similarity-based approach to leverage multi-cohort medical data on the diagnosis and prognosis of Alzheimer's disease

**DOI:** 10.1093/gigascience/giy085

**Published:** 2018-07-11

**Authors:** Hongjiu Zhang, Fan Zhu, Hiroko H Dodge, Gerald A Higgins, Gilbert S Omenn, Yuanfang Guan, the Alzheimer's Disease Neuroimaging Initiative

**Affiliations:** 1 Department of Computational Medicine and Bioinformatics, University of Michigan, 2017G Palmer Commons, 100 Washtenaw Avenue, Ann Arbor, MI, USA 48109; 2 Chongqing Key Laboratory of Big Data and Intelligent Computing, Chongqing Institute of Green and Intelligent Technology, Chinese Academy of Sciences, 266 Fangzheng Avenue, Shuitu Hi-tech Industrial Park, Shuitu Town, Beibei District, Chongqing, China 400714; 3 Michigan Alzheimer's Disease Center, University of Michigan, 2101 Commonwealth Blvd, Ann Arbor, MI, USA 48105; 4 Department of Neurology, University of Michigan, 1500 E. Medical Center Dr., 1914 Taubman Center SPC 5316, Ann Arbor, MI, USA 48109; 5 Layton Aging and Alzheimer's Disease Center and Department of Neurology, Oregon Health & Science University, 3181 S.W. Sam Jackson Park Road, L226, Portland, OR, USA 97239; 6 Department of Internal Medicine, University of Michigan, 3110 Taubman Center, SPC 5368, 1500 East Medical Center Drive, Ann Arbor, MI, USA 48109; 7 Department of Human Genetics, University of Michigan, 4909 Buhl Building, 1241 E. Catherine St., Ann Arbor, MI, USA 48109; 8 School of Public Health, University of Michigan, 1415 Washington Heights, Ann Arbor, MI, USA 48109; 9 Department of Electronic Engineering and Computer Science, Bob and Betty Beyster Building, 2260 Hayward Street, University of Michigan, Ann Arbor, MI, USA 48109

**Keywords:** Gaussian Process Regression, Alzheimer's Disease, Patient similarity network, Kernel method, Machine learning

## Abstract

**Motivation:**

Heterogeneous diseases such as Alzheimer's disease (AD) manifest a variety of phenotypes among populations. Early diagnosis and effective treatment offer cost benefits. Many studies on biochemical and imaging markers have shown potential promise in improving diagnosis, yet establishing quantitative diagnostic criteria for ancillary tests remains challenging.

**Results:**

We have developed a similarity-based approach that matches individuals to subjects with similar conditions. We modeled the disease with a Gaussian process, and tested the method in the Alzheimer's Disease Big Data DREAM Challenge. Ranked the highest among submitted methods, our diagnostic model predicted cognitive impairment scores in an independent dataset test with a correlation score of 0.573. It differentiated AD patients from control subjects with an area under the receiver operating curve of 0.920. Without knowing longitudinal information about subjects, the model predicted patients who are vulnerable to conversion from mild-cognitive impairment to AD through the similarity network. This diagnostic framework can be applied to other diseases with clinical heterogeneity, such as Parkinson's disease.

## Data Description

### Introduction

Alzheimer's disease (AD) is a heterogeneous, chronic, progressive disorder that leads to memory loss, cognitive impairment, psychiatric symptoms, and difficulties in daily activities [1, 2]. The disease affects more than 5.5 million people in the United States [3], and it is among the top 15 conditions with the greatest increase in global disease burden in the last decade [4, 5]. Currently, there is no cure for AD, but some treatments can provide symptomatic relief [6, 7]. Early diagnosis and treatment of the disease often offer cost benefits [1]. Unfortunately, it does not have a definitive marker test [8], and behavioral diagnosis is difficult in the early stage of the disease, limiting the potential for early treatment [9]. Sensitive and accurate diagnosis of the disease is greatly needed, preferably without requiring longitudinal data.

To improve dementia diagnosis, researchers have evaluated various ancillary diagnostic tests. Cerebrospinal fluid protein markers such as amyloid β (Aβ) and tau-protein (total τ-protein [T-τ] and phosphorylated τ-protein [P-τ]) have shown utility in AD diagnosis [10], and there are studies looking at other small molecule markers as well [11]. Previous reports have shown that estimates of tissue damage or loss from structural magnetic resonance imaging (MRI) are predictive of AD [12-16]. Other techniques such as positron emission tomography imaging of beta-amyloid plaques and tau aggregates have shown benefits as well [17-19]. The International Working Group and the United States National Institute on Aging–Alzheimer's Association Working Group proposed a series of diagnostic criteria for AD to better define clinical phenotypes and integrate biomarkers into the diagnostic process [20-25]. However, because of the disease heterogeneity, inexact nature of imaging tests, and cohort differences, quantitative standardization of these ancillary tests needs more calibration [26-30]. Although a biomarker classification scheme was proposed [31], behavior tests still play an important role in the diagnostic process [32].

Recent development in machine learning provides opportunities to deal with the problem from a different aspect. Successful applications of deep convolutional neural network in imaging segmentation enabled accurate and automatic brain segmentation using machine learning pipelines. Tools such as DeepNAT demonstrated accurate neuroanatomy segmentation [33, 34]. Researchers also have developed end-to-end diagnosis pipelines for AD diagnosis [35, 36]. However, one common critique is that these models are often hard to interpret [37]. Yet, the high performance of these machine learning methods is enlightening, and an approach that could combine the predictive power and the interpretability would be exciting.

In this study, we propose a quantitative approach to address the issue of heterogeneity in the diagnostic process. Instead of modeling markers explicitly, we explored similarity-based diagnostic modeling on patient data. Each incoming subject is compared to known individuals in the medical record database using a kernel function, and the Gaussian process method gives a diagnosis according to the similarity of medical conditions. Based on this idea, we developed a proof-of-concept AD diagnostic model that uses a combination of demographic, genetic, and MRI data, but without behavioral test features. To evaluate the method, our model was benchmarked on the Alzheimer's Disease Neuroimaging Initiative (ADNI) dataset using cross-validation tests. It was also tested in the Alzheimer's Disease Big Data DREAM Challenge on independent patient datasets and ranked highest among submitted methods [38, 39]. To further explore the potential of the similarity-based modeling, we enhanced the model on predicting AD progression among patients in the mild cognitive impairment (MCI) group and extended the approach to the diagnosis of Parkinson's disease.

## Methods

See Supplementary Information for details [40-47].

## Results

To address the problem of clinical heterogeneity, we developed a pipeline to match incoming subjects against known cases in the database and make a diagnosis that is more similar to those for individuals with similar conditions. This strategy was formulated as a Gaussian process. A Gaussian process model utilizes a kernel function to measure the similarity of the known individuals in the databases [48], weights all cases proportionally to the inverse of their similarity to the incoming subjects, and reports a weighted mean of all known diagnoses. Thus, the diagnostic prediction is biased toward the diagnoses of subjects with similar conditions. Various test results can be incorporated into the kernel function. Instead of asking for thresholds or value ranges for these tests, the model learns the distribution of these test results implicitly. The importance of different tests can also be adjusted quantitatively. With a fitting kernel function, a Gaussian process model is able to circumvent the clinical heterogeneity issue and make diagnosis accurately.

**Figure 1:**
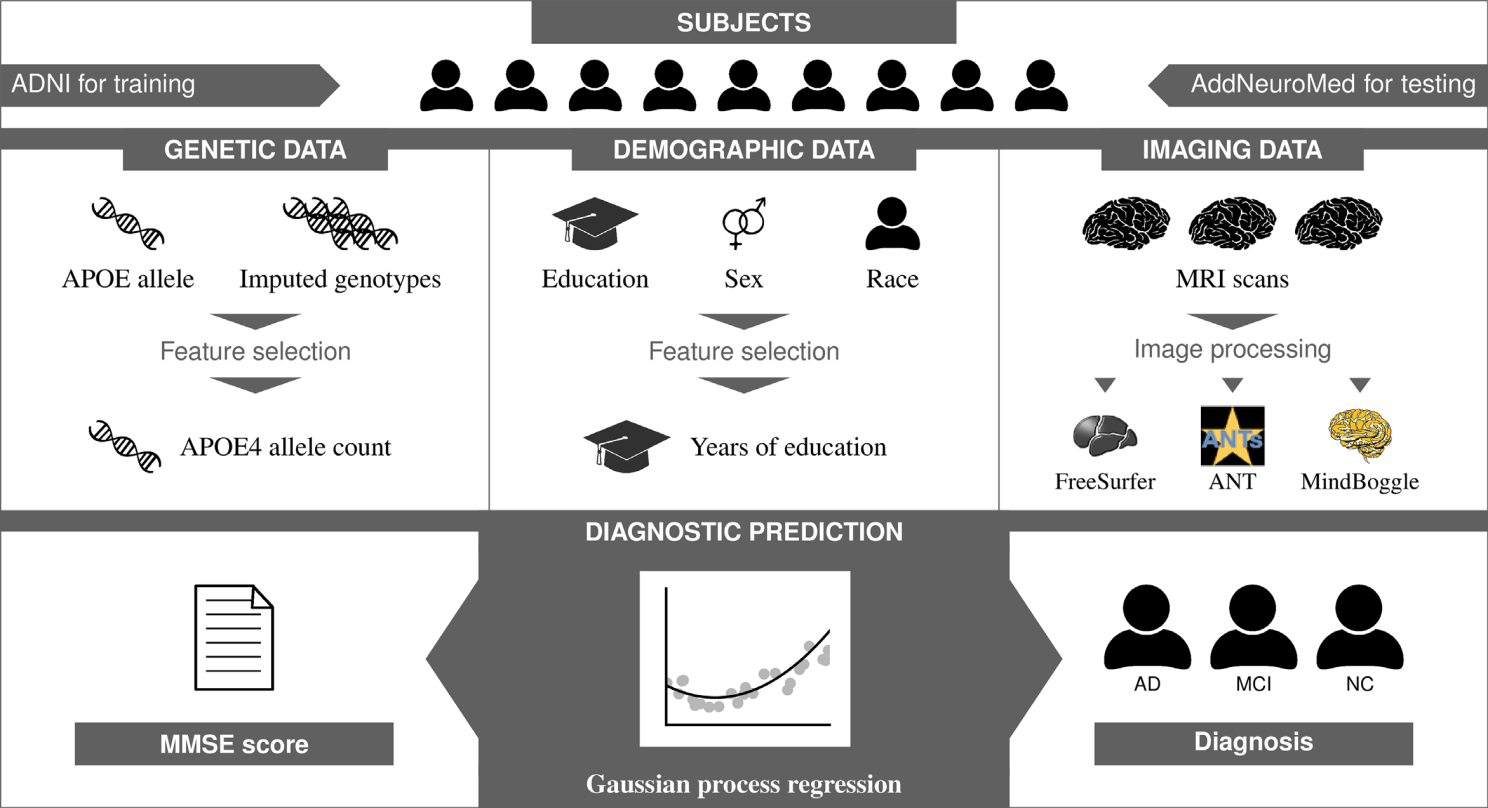
An overview of the AD diagnostic model. AD: Alzheimer's disease; CN: cognitive normal; MCI: mild cognitive impairment.

As numerous reports have revealed a high degree of heterogeneity in AD progression and clinical observations [29, 49, 50], we applied our approach to the diagnosis of AD as a proof of concept. An overview of our complete diagnostic model is shown in Fig. 1. For every subject, we collected demographic information, genetic data, and an MRI scan. MRI scans were preprocessed using FreeSurfer [43], ANT [44], and MindBoggle [45]. These programs labeled anatomic structures in the brain through image registration and performed surface- and voxel-based morphometric analyses, as well as output statistics about sizes, surface areas, and cortical thickness of these anatomical structures. We chose a limited set of features from these data in our kernel function, including education levels, Apolipoprotein E (APOE)allelic information, hippocampal volumes, amygdala volumes, and inferior lateral ventricle volumes. We also averaged the surface areas and volumes of multiple structures and incorporated them into the kernel function. Hippocampal volumes were chosen because they are the most correlated features and have been described repeatedly in the literature. The remaining features were chosen based on forward feature selection through cross-validation tests. We then used the Gaussian process regression model to predict the cognitive impairment in terms of Mini-mental state examination (MMSE) scores and to classify subjects into the cognitive normal, MCI, and AD groups.

Formulating population similarity as a Gaussian process brought us a valuable tool to investigate disease heterogeneity. By mapping known individuals in the training dataset into a hyperspace, we investigated the correlation between the kernel-transformed feature similarity and the cognition similarity at the individual level. We also explored the extensibility of the similarity modeling approach. By incorporating subject-level similarity network analysis, we enhanced the model to identify MCI patients who were at high risk of MCI-to-AD conversion. To further validate the performance of our approach on heterogeneous neurological diseases in general, we also evaluated our algorithm on Parkinson's disease data.

### Accurate prediction of cognitive impairment and diagnoses using similarity modeling

AD was chosen as a case study because of its reportedly high degree of heterogeneity at various levels. As a benchmark test, we first evaluated our approach on AD diagnosis and developed a model for estimating the severity of cognitive impairment and making a diagnosis using only baseline measurements without longitudinal information. We compared three major estimation strategies: linear modeling, decision tree modeling, and our similarity-based modeling. Linear models are well studied in many statistical models. They make strong assumptions about the linearity of factors. In this case, we tested linear regression models with different regularization schemes. Decision trees are similar to how humans make decisions; they iteratively divide the samples based on highly discriminative factors, such as age groups or hippocampal volume ranges, and make predictions for subgroups. We tested random forest, gradient boosting regression tree, and XGBoost regressor methods, which are widely used and have performed well in many machine learning studies. Similarity-based modeling corresponds to kernel methods in machine learning, where sample similarity is calculated using a devised distance function, and predictions are made accordingly. We tested support vector regressor (SVR) and Gaussian process regression model. All models were evaluated in a five-time five-fold cross-validation test. In a cross-validation test, the dataset was split into five parts. In each round, one part was withheld, and the models were trained with the remaining four parts. The performance of the models was then evaluated according to how well the models predicted on the withheld patient data. The test was repeated to reduce the variance of error estimation [47].

**Figure 2:**
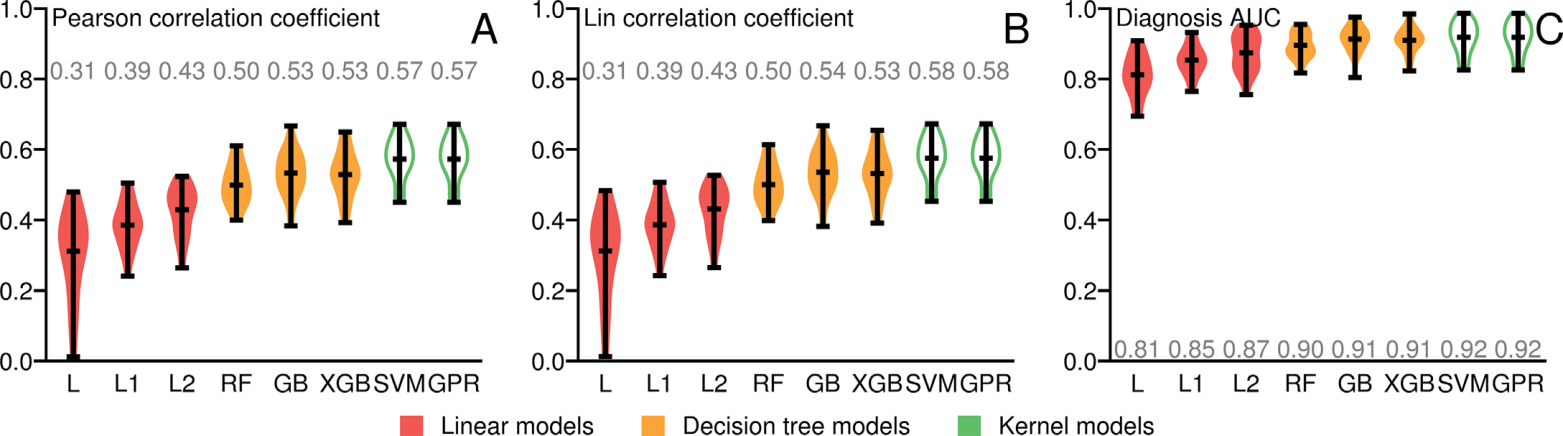
Violin plots of performance of different models estimated by cross-validation. **(A)** Performance of MMSE regression evaluated in terms of Pearson product-moment correlation coefficient. **(B)** Performance of MMSE regression evaluated in terms of Lin concordance correlation coefficient. **(C)** Performance of diagnostic predictions evaluated in terms of area under the curve. The average scores are labeled correspondingly. The final model performance is marked with a white body. GB: gradient boosting regression tree; GPR: Gaussian process regression with custom kernel; L: linear regression without regularization; L1: LASSO regression; L2: ridge regression; RF: random forest; SVM: kernel support vector regressor; XGB: XGBoost.

To show the performance of these three classes of diagnostic modeling, we benchmarked their performance in terms of cognition estimation and diagnosis accuracy on the ADNI1 dataset (Fig. 2). We evaluated the accuracy of cognitive impairment in terms of Pearson correlation coefficient and Lin concordance correlation coefficient. The two metrics focus on different aspects. A high Pearson correlation coefficient suggests that the prediction can be well aligned with the observed values linearly, while a high Lin concordance correlation coefficient indicates the similarity between the intragroup distributions of two datasets [51]. In our tests, similarity-guided methods outperformed other methods by a large margin in both metrics. The Gaussian process method marginally outperformed the SVR model and became the best prediction model.

We also evaluated the diagnosis accuracy of all models. We tested making a diagnosis based on the cognitive impairment predictions and evaluated the performance in terms of the area under curve (AUC) of normal against AD/MCI classification. The Gaussian process method again outperformed other methods. We then further optimized the parameters of the model (Supplementary Fig. S1). The final model was also tested on a hidden dataset from AddNeuroMed in the DREAM Challenge and predicted the cognition scores with a Pearson correlation coefficient of 0.573. Its performance on an independent multisectional study was consistently good in comparison to its performance in the cross-validation tests. We then moved on to study individual similarity estimation, subtype identification, and extensibility of the optimized similarity model.

### Subject-level analysis on the severity of cognitive impairment using kernel methods

**Figure 3:**
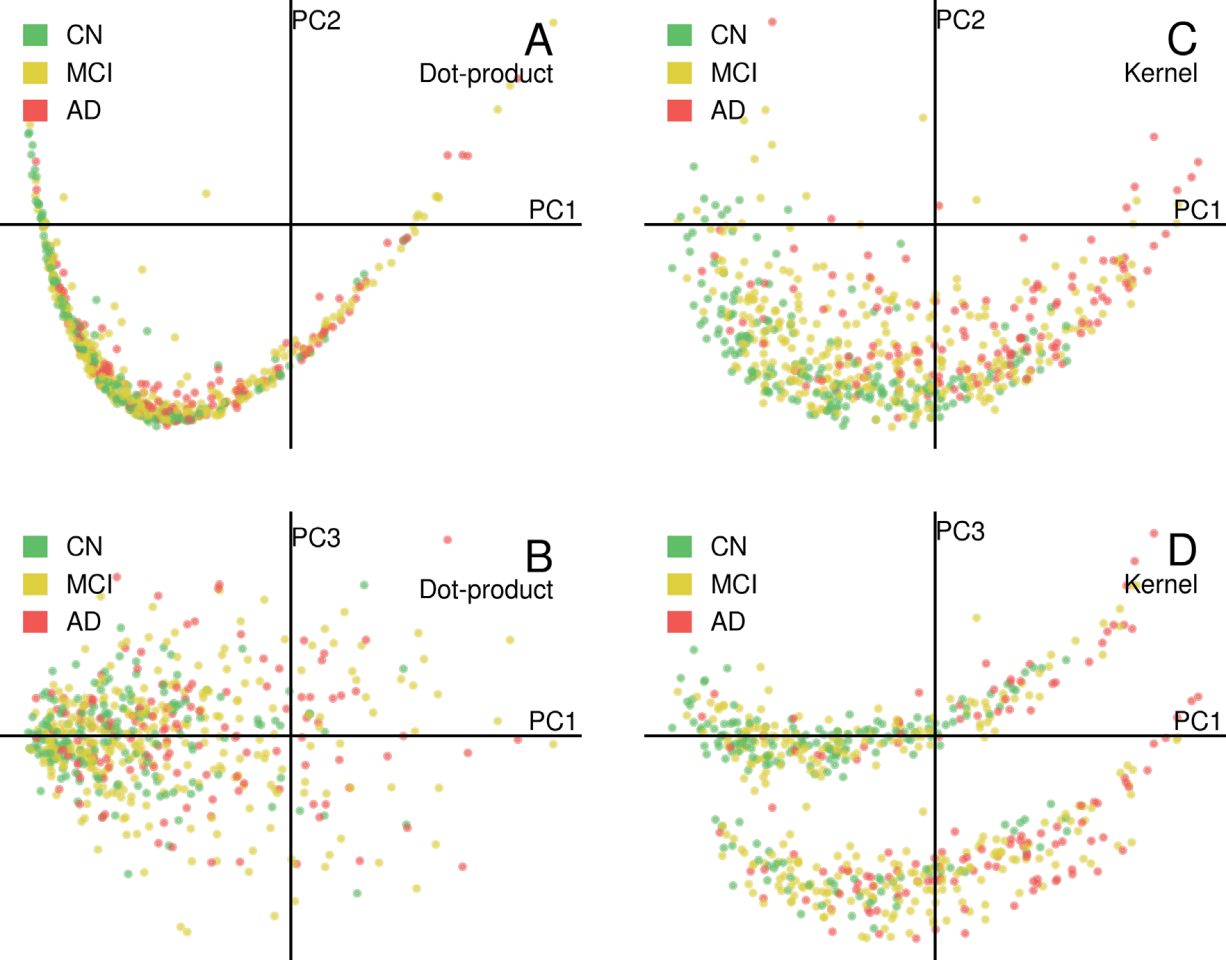
PCA over the dot-product similarity matrix **(A and B)** and the custom kernel similarity matrix **(C and D)**. PCA on kernel matrix revealed patterns of different disease progressions in the transformed feature space.

Working with heterogeneous data from a multisectional study poses a challenge to data modeling. We addressed the heterogeneity issue by matching prediction targets to subjects with similar conditions using a kernel function and thus avoided specifying explicit thresholds or ranges for biomarkers across different cohorts. Since the similarity model is the core of our strategy, we assessed the effectiveness of our similarity model on the ADNI1 dataset. We performed principal component analysis (PCA) over two different similarity matrices, one calculated from a dot-product similarity (which is equivalent to linear regression model) and the other from our custom kernel. With the custom kernel, PCA showed a strong correlation between AD progression and transformed MRI features (Fig. 3C). It also clustered samples according to risk factors such as APOE ε4 allelic count (Fig. 3D). Such a pattern was not clearly observed from the dot-product similarity model (Fig. 3A and 3B). Visualization of the spatial distribution of samples before and after kernel transformation suggested that the kernel might extract a strong signal and estimate individual similarity well.

To validate our hypothesis from the above analysis, we then compared the results of the similarity functions to the similarity of cognition of individuals. For each sample, we calculated its feature-wise similarity to all other samples using both dot-product and custom kernel functions and then computed dissimilarity correlation scores (DCSs). DCSs quantify the correlation between cognitive impairment differences and the reciprocals of their similarity (Supplementary Fig. S2). An effective similarity measurement is expected to have a high DCS, showing that feature-wise dissimilarity correlates with diagnostic dissimilarity. The kernel transformation was significantly better than random permutation (1-sided *t*test *P* < 0.001) and significantly improved DCS over the linear similarity model (1-sided *t*test *P* < 0.001). Both visualization and quantitative analysis suggested that the kernel methods gave good estimation on cognition similarity between individuals with various clinical features.

### Applications to AD progression and Parkinson's disease diagnostic predictions

Beyond estimating the cognitive impairment and predicting AD diagnoses, our model can be extended to other scenarios. For example, an important aspect in AD study is identifying MCI patients who might soon convert to AD. Here, we extended our diagnostic model to a progression prediction model with only baseline measurements by incorporating network analysis. To investigate the specificity of our approach in regards to the vulnerable subgroup, we first looked into the predictions of our diagnostic model. (See Supplementary Table S2 for the confusion matrix of our prediction evaluation.) While our approach achieved a high AUC in diagnosing MCI/AD subjects against normal subjects, 110 of 296 MCI patients were misclassified as AD patients. In this case, our diagnostic model made predictions based on patient similarity and considered these patients to be more similar to AD patients than other MCI patients. Thus, we compared this result to the longitudinal data present in the ADNI database. Of 110 MCI subjects who were predicted as AD patients, 75 (68%) eventually converted to AD in the later follow-up examinations during four years. The AD predictions were significantly biased toward patients who later converted to AD (Fisher exact test *P* < 0.001).

**Figure 4:**
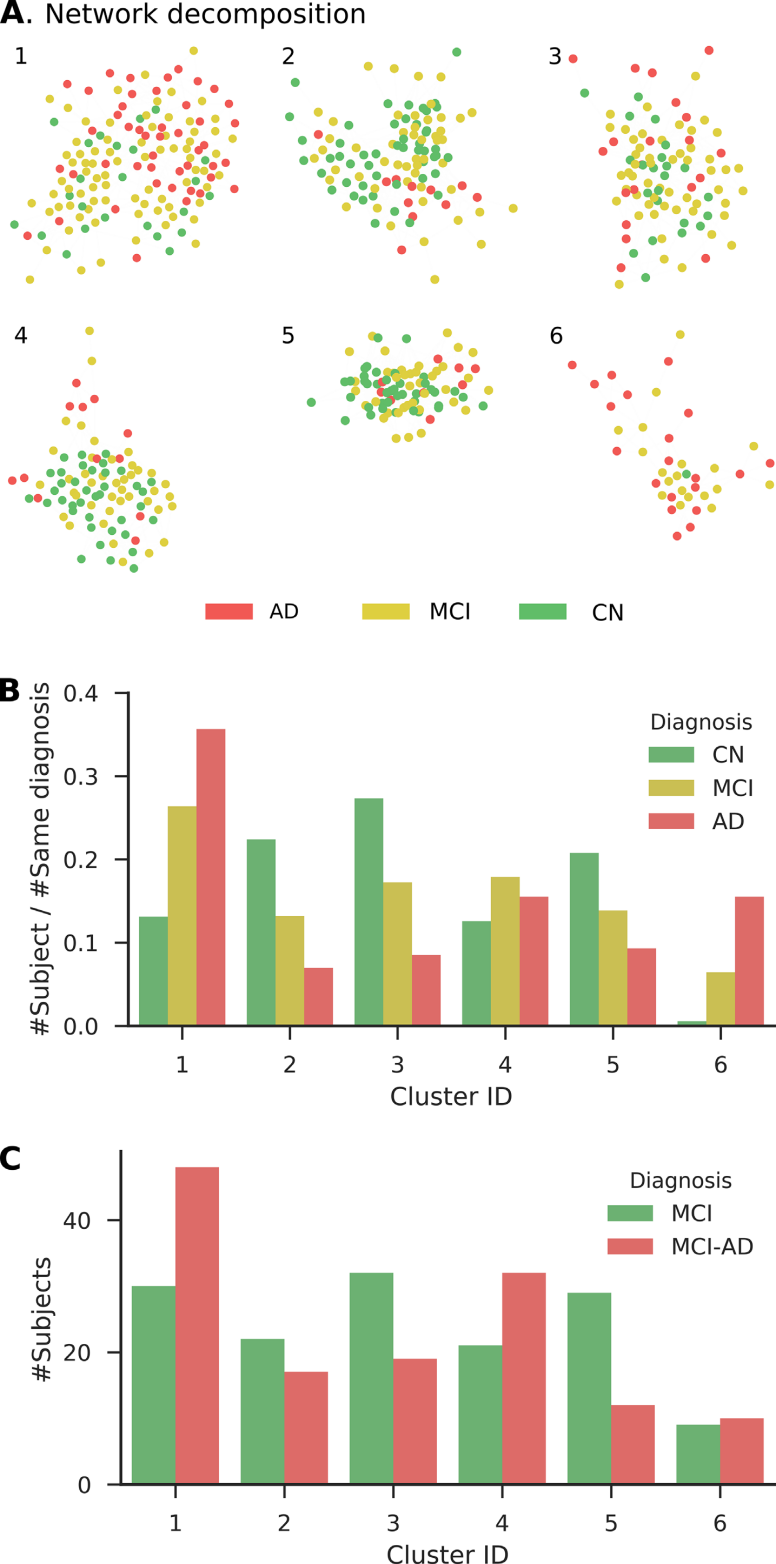
Similarity networks of subjects in the ADNI dataset, decomposited by Girvan-Newman algorithm. **(A)** Visualization of the clusters. Subjects are colored according to baseline diagnosis. **(B)** The distribution of three diagnostic types in two clusters. **(C)** The distribution of MCI-to-AD conversion/nonconversion subjects in two clusters.

The uneven distribution (of misclassification cases), provided that our algorithm predicts diagnosis based on patient similarity, suggests the correlation between the similarity of these MCI patients to AD and normal subjects and the disease progression of these MCI patients. More specifically, MCI patients whose conditions resemble those of AD patients might face higher risks of AD conversion, while other MCI patients might be less likely to develop AD. To test the hypothesis, we analyzed the patient similarity network. We built a network that connects subjects in the training dataset with edges. The weights of the edges are the similarity between connecting subjects calculated by the kernel function in our algorithm. Under our hypothesis, highly weighted edges would associate MCI patients who converted later to AD more closely than normal subjects in the network. To analyze the connective patterns of highly weighted edges, we first trimmed the network by filtering out the lowest 97.5% weighted edges and then applied the Girvan-Newman community clustering algorithm (as implemented in clusterMaker2, a Cytoscape plugin). We chose the threshold by comparing the modularity of the final clustering results against that of trimmed and clustered random networks; the threshold level of 97.5% achieved the most significant difference. The Girvan-Newman clustering algorithm decomposed the trimmed network into 10 clusters (Fig. 4). We dropped the smallest four clusters out of the analysis, each of which has fewer than 10 subjects. Among the remaining clusters, clusters 1, 3, and 6 contained more normal subjects than AD patients, while clusters 2, 4, and 5 contained more AD patients. Based on the clustering results, we directly predict that those MCI patients in clusters 1, 3, and 6 have low risks of disease progression and that those in the other clusters have high risks. A Fisher exact test on these subjects confirmed the discriminative power (p = 0.0001). Despite not including any longitudinal data, our model successfully captured the properties of the subpopulation that is vulnerable to MCI-to-AD conversion. It demonstrated the effectiveness of the similarity function we adopted in the prediction model.

**Figure 5:**
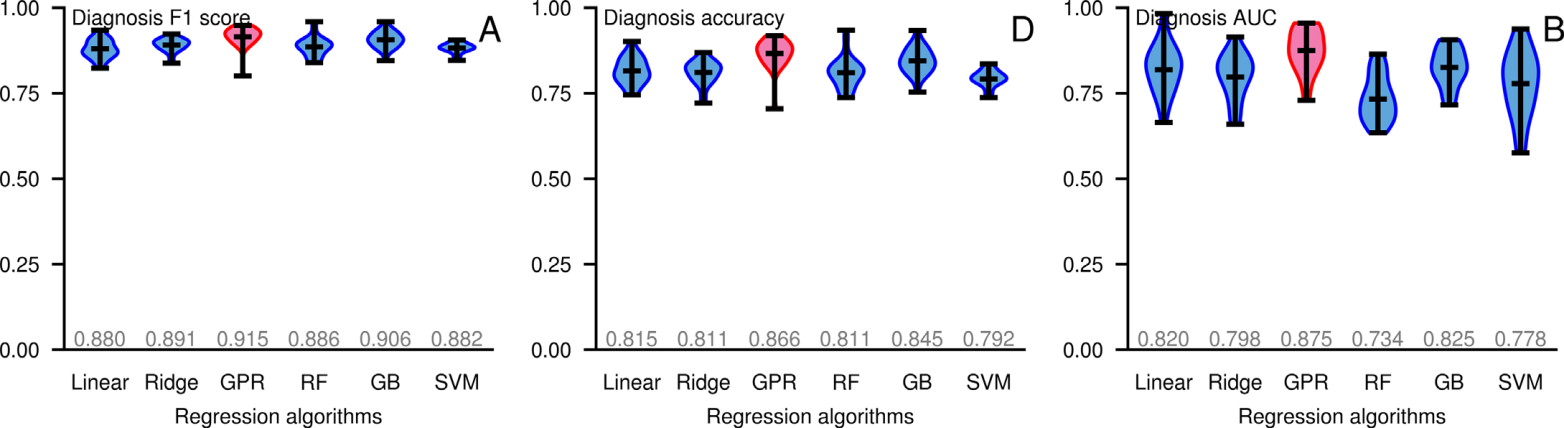
Violin plots of performance of different diagnostic models estimated on Parkinson's disease dataset by cross-validation. Performance is evaluated in terms of F1 scores, ratio of correct predictions, and AUC.The average scores are labeled correspondingly. GB: gradient boosting regression tree; GPR: Gaussian process regression with custom kernel; Linear: linear regression without regularization; RF: random forest; Ridge: ridge regression; SVM: kernel support vector regressor.

The idea of using subjects with similar conditions to guide diagnosis is not specific to AD, and the model we proposed in this study can be applied to other diseases as well. To demonstrate its extensibility, we built a Gaussian process diagnosis model for Parkinson's disease. The model was trained on the Parkinson's Progression Markers Initiative dataset [46]. The MRI images were processed using FreeSurfer to extract numeric features such as surface areas and volumes. We included ages and genders as features, together with areas and volumes of brain anatomic structures estimated by FreeSurfer. We performed repeated 5-fold cross validation on 302 subjects in the dataset. The Gaussian process model achieved an AUC of ∼0.88 (Fig. 5, Supplementary Fig. S3). Thus, for heterogeneous neurological diseases other than AD, the similarity-based diagnostic strategy can give accurate diagnostic predictions as well.

## Discussion

In this study, we show that similarity-based diagnostic modeling is an effective approach to deal with heterogeneous diseases and nonlinear clinical data. The modeling focuses on subjects with similar conditions and uses their diagnoses to guide our decisions. We tested the method in AD and achieved very good performance in estimating the severity of cognitive impairment indicated by MMSE scores and predicting diagnoses. Specifically for MCI patients, the model even captured the differences between those who would later convert to AD and those who would not. We also tested the method in Parkinson's disease to show the generality of the modeling approach.

The idea of similarity modeling applied here to disease diagnosis echoes recent studies on population modeling. Various social network studies have demonstrated that local similarity predicts various characteristics of individuals [52-54]. Adapting this idea from social network settings, previous studies found that the similarity approach can be used to estimate disease risks [55-57]. Here, we focused on individual diseases where heterogeneity hinders accurate diagnosis. Previously we showed that a Gaussian process is effective in handling medical datasets of limited samples [58, 59], and recent reports indicate that it can also be extended to larger-scale studies [60].

While the idea of similarity modeling has been applied to many topics, our method still needed to solve four major challenges specific to diagnostic modeling: it must deal with heterogeneous nonlinear data, remain interpretable in medical contexts, provide insights into disease progression, and be effective on different cohorts, new assays, and even other diseases. A method that achieves these goals would bridge the gap between conventional diagnostic models and advanced machine learning models in terms of predictive power, interpretability, perceptiveness, and flexibility. We paid close attention to these four focuses throughout the study:

First, our similarity approach is a nonparametric approach that does not assume the underlying distributions of risk factors [61]. Commonly adopted methods often assume independence and a linear relationship between features for simplicity and work adequately for some cases. Yet, for diseases that manifest heterogeneous phenotypes, such an assumption can be incorrect, and a more complicated model may be necessary [62]. This is particularly true when dealing with multi-cohort datasets, where batch effects can further obscure the relationship between observed measurements and clinical outcomes [63]. In comparison to many commonly used regression methods, our nonparametric approach is more flexible in dealing with nonlinear data. The kernel function brings more accurate modeling and stronger predictive power.

Second, our approach is a well-studied statistical model that, unlike many other advanced machine learning models, can be easily interpreted. Many advanced machine learning models have been developed for diagnosis of AD [64], especially various deep neural network models [64, 65-67]. While most of these models show great performance in diagnostic classification, interpreting deep learning models remains a hard problem [68]. Uninterpretable models in medical applications can be undesirable in some cases [37, 69]. A Gaussian process, on the other hand, is a well-studied statistical model and takes an intuitive approach. The prediction process can be performed with little human intervention, and yet physicians can still read the estimated confidence interval from Gaussian process regression to judge how confident the diagnosis is. The similarity of individual subjects reported from the model also provides the reason behind the prediction. A bonus point to our approach is that it is possible to combine the interpretability of Gaussian process and the power of deep neural network together through techniques such as ensemble model and stack generalization [70].

Third, our method identifies subjects who manifest different disease progression rates through the pattern of the similarity network. Most excitingly, we found that our model accomplished this when fed only baseline information and lacked any longitudinal information of training subjects. Previous research showed that MCI patients who would later convert to AD show traits that are similar to those of AD patients in their baseline measurements [71, 72]. It allows our model to capture features to predict disease progression. The network provides a different view of the subjects and can be a powerful tool for researchers to investigate the heterogeneity of disease progression at a population level [57, 73]. Diagnosis of other similar diseases that show heterogeneity in disease progression could benefit from the application of our network analysis approach.

Finally, our method can be easily transferred to different cohorts, assays, and diseases. In the Alzheimer's Disease Big Data DREAM Challenge, our model has been tested on AddNeuroMed, an independent multi-cohort AD study focusing on European subjects [42] and achieved consistently high performance [38]. The feature input to our method allows incorporation of imaging data, genotype information, biochemical marker assays, and many other tests. Recent studies on AD risk factors can be incorporated into the similarity function [11, 74, 75]. Here, we have shown its application to the diagnosis of Parkinson's disease, and previously we developed Gaussian process models for other diseases [58]. The model demonstrates extensibility and flexibility in dealing with different datasets without compromising its state-of-the-art predictive power in diagnostic prediction.

While our approach dealt with the four challenges listed above, there is still room for improvement. While our AD model achieved promising results, it was developed under rather harsh settings (required by the challenge for competition fairness). The DREAM Challenge chose MMSE as one of the prediction targets of the competition, yet studies have shown MMSE is not an indicator specific enough for AD [76, 77]; for the challenge, no other disease-specific indicators were available. In addition, the MRI images were automatically processed by programs without manual intervention, and recent studies suggest the accuracy of this auto-labeling pipeline can be further improved [78, 79], which would boost the performance. Fortunately, these limitations can be avoided or solved in real-world applications, in which case our method would achieve even better results. Further applications to different cohorts, clinical tests, and diseases will be of interest. On the computational side, kernel matrix calculation requires comparing all pairs of samples. The time and memory complexity grows quadratically when the number of samples increases. Researchers have found approximation of kernel calculations for large datasets, but their effects on prediction accuracy of our model needs further investigation.

## Conclusions

We presented a novel computational approach that estimates the cognitive impairment of AD patients. The method calculates the similarity between subjects using structural MRI data and other clinical measurements from multisectional studies and predicts cognitive impairment with biases toward patients with high similarity. The method, fed with ADNI1 data, demonstrated its state-of-the-art predictive power on disease diagnosis. The idea of relating incoming subjects to known cases of similar conditions allows more specialized diagnosis. Without any information about disease progression, unsupervised similarity network analysis predicted patients at high risk of MCI-to-AD conversion. The effectiveness of this similarity-based approach was validated on an independent cohort and tested on Parkinson's disease, another common, heterogeneous neurological disease. The promising performance of our model suggests that not only can it be an alternative approach to establishing quantitative diagnostic criteria, it also represents an attractive tool for researchers to study disease progression.

## Availability of supporting data

The original competition code, together with demonstration data, are available at https://www.synapse.org/#!Synapse:syn2527678/wiki/69937. Additional code is available at https://github.com/GuanLab/2014_AD_Sup. Data are also available in the *GigaScience* GigaDB repository [80].

## Additional files

**Figure S1:** Violin plots of performance of different models estimated by cross-validation. Performance of MMSE regression is evaluated in terms of Pearson product-moment correlation coefficient (A and D) and Lin's concordance correlation coefficient (B and E). Performance of diagnostic predictions is evaluated in terms of AUC (C and F). The average scores are labelled correspondingly. The models chosen for the final submissions are marked with red body. A, B, & C. The performance of different noise parameters a in GPR. D, E, & F. The performance of different preprocessors for MRI data (tested with GPR regressors).

**Figure S2:** Kernel density estimation of the distribution of dissimilarity correlation score (DCS) of both linear and kernel similarity models (the higher DCS the better).

**Figure 3:** Receiver operating characteristic curve of 5-fold cross validation on Parkinson's disease dataset.

**Table S1:** The confusion matrix of the Gaussian process diagnostic model's prediction on ADNI training dataset

**Table S2:** A summary of the ADNI training dataset provided by the competition.

**Table S3:** A summary of the AddNeuroMed testing datasets provided by the competition.

## Abbreviations

AD: Alzheimer's Disease; ADNI: Alzheimer's Disease Neuroimaging Initiative; APOE: Apolipoprotein E; AUC: area under curve; DCS: dissimilarity correlation scores; MCI: mild-cognitive-impairment; MMSE: Mini-mental state examination MRI: Magnetic resonance imaging; PCA: principal component analysis; SVR: support vector regressor.

## Competing interests

The authors declare that they have no competing interests.

## Supplementary Material

GIGA-D-17-00279_Original_Submission.pdf

GIGA-D-17-00279_Revision_1.pdf

Response_to_Reviewer_Comments_Original_Submission.pdf

Reviewer_1_Report_(Original_Submission) -- tavros Dimitriadis I Dimitriadis 12/13/2017 Reviewed

Reviewer_1_Report_(Revision_1) -- tavros Dimitriadis I Dimitriadis 4/26/2018 Reviewed

Reviewer_2_Report_(Original_Submission) -- Chao Huang 1/13/2018 Reviewed

Reviewer_2_Report_(Revision_1) -- Chao Huang 5/25/2018 Reviewed

Supplemental Files
