# Supplementary material for: A similarity-based approach to leverage multi-cohort medical data on the diagnosis and prognosis of Alzheimer's disease
Source: Gigascience. 2018 Jul 11;7(7):giy085. doi: 10.1093/gigascience/giy085 (PMC6054197; doi:10.1093/gigascience/giy085)
Supplement: GIGA-D-17-00279_Revision_1.pdf [file giy085_giga-d-17-00279_revision_1.pdf]

GigaScience

A similarity-based approach to leverage multi-cohort medical data on the diagnosis and prognosis of Alzheimer's disease

--Manuscript Draft--

**Manuscript Number:**	GIGA-D-17-00279R1
**Full Title:**	A similarity-based approach to leverage multi-cohort medical data on the diagnosis and prognosis of Alzheimer's disease
**Article Type:**	Research
**Funding Information:**	National Institute on Aging (P30AG053760) — Dr. Yuanfang Guan<br>Division of Biological Infrastructure (1452656) — Dr. Yuanfang Guan<br>Alzheimer's Association (US) (Cross-disease brain image modeling) — Dr. Yuanfang Guan
**Abstract:**	Motivation: Heterogeneous diseases such as Alzheimer's disease manifest a variety of phenotypes among populations. Early diagnosis and effective treatment offer cost-benefits. Many studies on biochemical and imaging markers have shown potential promise in improving diagnosis, yet establishing quantitative diagnostic criteria for ancillary tests remains challenging.<br>Results: We have developed a similarity-based approach that matches individuals to subjects with similar conditions. We modeled the disease with a Gaussian process, and tested the method in the Alzheimer's Disease Big Data DREAM Challenge. Ranked the highest among submitted methods, our diagnostic model predicted cognitive impairment scores in an independent dataset test with a correlation score of 0.573. It differentiated Alzheimer's disease patients from control subjects with an area under receiver operating curve of 0.920. Without knowing longitudial information about subjects, the model predicted patients that are vulnerable to MCI-to-AD conversion through the similarity network. This diagnostic framework can be applied to other diseases with clinical heterogeneity, such as Parkinson's disease.
**Corresponding Author:**	Yuanfang Guan<br><br>UNITED STATES
**Corresponding Author Secondary Information:**	
**Corresponding Author's Institution:**	
**Corresponding Author's Secondary Institution:**	
**First Author:**	Hongjiu Zhang
**First Author Secondary Information:**	
**Order of Authors:**	Hongjiu Zhang
	Fan Zhu
	Hiroko H Dodge
	Gerald A Higgins
	Gilbert S Omenn
	Yuanfang Guan
**Order of Authors Secondary Information:**	
**Response to Reviewers:**	Dear Dr. Scott Edmunds,<br><br>We write to submit our revision of the manuscript titled “A similarity-based approach to leverage multi-cohort medical data on the diagnosis and prognosis of Alzheimer's

Powered by Editorial Manager® and ProduXion Manager® from Aries Systems Corporation

disease". We have addressed all of the reviewers' questions in the manuscript. The responses and corresponding changes are attached at the end of the email.

We uploaded to Github all the code associated with the new experiments suggested by reviewers, as well as annotated IPython notebooks of all experiments in the manuscript. The data availability has been changed accordingly. We ensure that readers can reproduce all our results with the data mentioned in the "data availability" section.

We appreciate all reviewer's constructive suggestions. Thank you for your interest in our work.

Yours sincerely,

Yuanfang Guan
Department of Computational Medicine and Bioinformatics, Palmer Commons
100 Washtenaw Avenue, Ann Arbor, MI 48109-2218
University of Michigan

===

Reviewer #1:

1) Authors used SVM as a regressor candidate algorithm.
Is SVM or SVR ?

Thanks for pointing this out. The regression algorithm is SVR. We have changed all mentioned SVM to SVR.

2) In the examination of the distribution of MCI-to-AD converted patients using similarity network analysis, they constructed a network based on their kernel approach estimated between every pair of subjects with the adopted features.
Then, they applied a density threshold by keeping the 5% of the strongest connections and finally GN algorithm is applied.

It is better to optimize the theshold scheme based on the p-values of the original Q quality values of the clustering algorithm and the one derived by randomize the original similarity matrix by e.g. 1000 times.
BCT contains algorithms for the randomization of a matrix be keeping the strength and the degree of each node.

Thanks for your suggestions. We followed your suggestion. The result is described in Page 8:

"The uneven distribution" (of misclassification cases), "provided that our algorithm predicts diagnosis based on patient similarity, suggests the correlation between the similarity of these MCI patients to AD and normal subjects and their disease progression. More specifically, MCI patients whose conditions resemble those of AD patients might face higher risks of AD conversion, while other MCI patients might be less likely to develop AD. To test the hypothesis, we analyzed the patient similarity network. We built a network that connects subjects in the training dataset with edges. The weights of the edges are the similarity between connecting subjects calculated by the kernel function in our algorithm. Under our hypothesis, highly weighted edges would associate MCI patients that converted later to AD patients more closely than normal subjects in the network. To analyze the connective patterns of highly weighted edges, we first trimmed the network by filtering out the lowest 97.5% weighted edges and then applied Girvan-Newman community clustering algorithm (as implemented in clusterMaker2, a Cytoscape plugin). We chose the threshold by comparing the modularity of the final clustering results against that of trimmed and clustered random networks, and the threshold level of 97.5% achieved the most significant difference (Supplementary Table 4). Girvan-Newman clustering algorithm decomposed the trimmed network into 10 clusters (Fig 4). We dropped the smallest 4 clusters out of the analysis, each of which has less than 10 subjects. Among the remaining clusters, Cluster 1, 3, and 6 contained more normal subjects than AD patients, while Cluster 2,

Powered by Editorial Manager® and ProduXion Manager® from Aries Systems Corporation

	4, and 5 contains more AD patients. Based on the clustering results, we directly predict that those MCI patients in Cluster 1, 3, and 6 have low risks in disease progression, as well as those in the other clusters have high risks. A Fisher exact test on these subjects confirmed the discriminative power (p = 0.0001). Despite not including any longitudinal data, our model successfully captured the properties of the subpopulation which is vulnerable to MCI-to-AD conversion. It demonstrated the effectiveness of the similarity function we adopted in the prediction model."<br><br>The method description is added to the Supplementary Information as a new section:<br><br>"We constructed a subject-level similarity network. The network connects all training subjects with edges, and the weights of the edges are calculated by the kernel function k. The network is then trimmed to keep only highest weighted edges. In order to choose the optimal filter threshold, we tested keeping top 0.5%, 1%, 2.5%, 5%, and 10% weighted edges. For each threshold, we applied the community clustering and evaluated their modularity in terms of modularity Q scores. At the same time, we generated 100 random networks for comparison by randomly shuffling edge weights in the original network, trimmed and clustered these networks using all tested thresholds, and calculated their modularity scores. We then determined the significance for original Q scores at each threshold level by counting how many times the randomized Q scores are larger than the original scores. We chose the most significant threshold level that has least number of higher Q scores from clustering random networks. We then apply Girvan-Newman community clustering algorithm on the trimmed network, performed by GLay clustering in clusterMaker2, a Cytoscape plugin."<br><br>3) I would like to see more comments regarding the limitation of the whole process including gaussian distribution and kernel approaches.<br><br>Thanks for the question. We appended the following discussion to the last paragraph of the Discussion section (Page 12):<br><br>"On the computational side, kernel matrix calculation requires comparing all pairs of samples. The time and memory complexity grows quadratically when the number of samples increases. Researchers have found approximation of kernel calculations for large datasets, but their effects on prediction accuracy of our model needs further investigation."<br><br>===<br><br>Reviewer #2:<br><br>1) In this paper, for the ADNI data analysis, you choose the APOE4, education levels, and several imaging features. Is there any guideline to choose these covariates?<br><br>Thanks for the question. We have extended the method section in regards of feature engineering. We also added a short description on this part in the Results section (Page 3, Line 117-119):<br><br>"Hippocampal volumes are chosen because they are the most correlated features and have been described repeatedly in the literature. The remaining features are chosen based on forward feature selection through cross-validation tests."<br><br>2. When you compare your GPR model with other prediction models, could you also try XG-Boost as well? As I know, XG-Boost currently is very popular and of great power in machine learning.<br><br>Thank you for the suggestion. We have included the result of XG-Boost into the comparison. The Figure 2 is updated. Given that XG-Boost is similar to general gradient boosting regression tree (except for pruning and binning details), the performance of XG-Boost is very close to gradient boosting regression tree.
**Additional Information:**	
**Question**	**Response**

Powered by Editorial Manager® and ProduXion Manager® from Aries Systems Corporation

Are you submitting this manuscript to a special series or article collection?	No
**Experimental design and statistics**<br><br>Full details of the experimental design and statistical methods used should be given in the Methods section, as detailed in our Minimum Standards Reporting Checklist. Information essential to interpreting the data presented should be made available in the figure legends.<br><br>Have you included all the information requested in your manuscript?	Yes
**Resources**<br><br>A description of all resources used, including antibodies, cell lines, animals and software tools, with enough information to allow them to be uniquely identified, should be included in the Methods section. Authors are strongly encouraged to cite Research Resource Identifiers (RRIDs) for antibodies, model organisms and tools, where possible.<br><br>Have you included the information requested as detailed in our Minimum Standards Reporting Checklist?	Yes
**Availability of data and materials**<br><br>All datasets and code on which the conclusions of the paper rely must be either included in your submission or deposited in publicly available repositories (where available and ethically appropriate), referencing such data using a unique identifier in the references and in the "Availability of Data and Materials" section of your manuscript.<br><br>Have you have met the above requirement as detailed in our Minimum	Yes

Standards Reporting Checklist?	

Powered by Editorial Manager® and ProduXion Manager® from Aries Systems Corporation

Manuscript
Click here to access/download;Manuscript;Manuscript(2)(1).docx
Click here to view linked References

A similarity-based approach to leverage multi-cohort medical data on the diagnosis and prognosis of Alzheimer's disease

Hongjiu Zhang[1,†], Fan Zhu[1,2,†], Hiroko H. Dodge[3,4,5], Gerald A. Higgins[1], Gilbert S. Omenn[1,6,7,8], Yuanfang Guan[1,6,9,*], for Alzheimer’s Disease Neuroimaging Initiative[¶]

[1] Department of Computational Medicine and Bioinformatics, University of Michigan, Ann Arbor, MI, USA 48109
[2] Baidu LLC USA, Sunnyville, CA, USA 94089
[3] Michigan Alzheimer’s Disease Center, University of Michigan, Ann Arbor, MI, USA 48109
[4] Department of Neurology, University of Michigan, Ann Arbor, MI, USA 48109
[5] Layton Aging and Alzheimer’s Disease Center and Department of Neurology, Oregon Health & Science University, Portland, OR, USA 97239
[6] Department of Internal Medicine, University of Michigan, Ann Arbor, MI, USA 48109
[7] Department of Human Genetics, University of Michigan, Ann Arbor, MI, USA 48109
[8] School of Public Health, University of Michigan, Ann Arbor, MI, USA 48109
[9] Department of Electronic Engineering and Computer Science, University of Michigan, Ann Arbor, MI, USA 48109

[†] Zhang and Zhu equally contributed to the work.
[*] To whom correspondence should be addressed: gyuanfan@umich.edu, ORCID: 0000-0001-8275-2852
[¶] Data used in preparation of this article were obtained from the Alzheimer’s Disease Neuroimaging Initiative (ADNI) database (http://adni.loni.usc.edu). As such, the investigators within ADNI contributed to the design and implementation of ADNI and/or provided data but did not participate in analysis or writing of this report. A complete listing of ADNI investigators can be found at: http://adni.loni.usc.edu/wp+content/up+loads/how_to_apply/ADNI_Acknowledgement_List.pdf.

Abstract

Motivation: Heterogeneous diseases such as Alzheimer's disease manifest a variety of phenotypes among populations. Early diagnosis and effective treatment offer cost-benefits. Many studies on biochemical and imaging markers have shown potential promise in improving diagnosis, yet establishing quantitative diagnostic criteria for ancillary tests remains challenging.

Results: We have developed a similarity-based approach that matches individuals to subjects with similar conditions. We modeled the disease with a Gaussian process, and tested

the method in the Alzheimer's Disease Big Data DREAM Challenge. Ranked the highest among submitted methods, our diagnostic model predicted cognitive impairment scores in an independent dataset test with a correlation score of 0.573. It differentiated Alzheimer's disease patients from control subjects with an area under receiver operating curve of 0.920. Without knowing longitudial information about subjects, the model predicted patients that are vulnerable to MCI-to-AD conversion through the similarity network. This diagnostic framework can be applied to other diseases with clinical heterogeneity, such as Parkinson's disease.

Data Description

The original competition code, together with demonstration data, is available at https://www.synapse.org/#!Synapse:syn2527678/wiki/69937. Additional code is available at https://github.com/GuanLab/2014_AD_Sup .

Introduction

Alzheimer's disease (AD) is a heterogeneous chronic progressive disorder that leads to memory loss, cognitive impairment, psychiatric symptoms, and difficulties in daily activities [1,2]. The disease affects over 5.5 million people in the United States [3], and it is among top 15 conditions with the greatest increase in global disease burden in the last decade [4,5]. Currently, there is no cure for Alzheimer's disease, but some treatments can provide symptomatic relief [6,7]. Early diagnosis and treatment of the disease often offer cost-benefits [1]. Unfortunately, it does not have a definitive marker test [8], and behavioral diagnosis of the disease is difficult in the early stage of the disease, limiting the potential for early treatment [9]. Sensitive and accurate diagnosis of the disease is greatly needed, preferably without requiring longitudinal data.

To improve dementia diagnosis, researchers have evaluated various ancillary diagnostic tests. Cerebrospinal fluid protein markers such as amyloid β (Aβ) and tau-protein (total τ-protein [T-τ] and phosphorylated τ-protein [P-τ]) have shown utility in Alzheimer's disease diagnosis [10], and there are studies looking at other small molecule markers as well [11]. Previous reports have shown that estimates of tissue damage or loss from structural magnetic resonance imaging (MRI) are predictive of Alzheimer's disease [12–16]. Other techniques such as positron emission tomography imaging of beta-amyloid plaques and tau aggregates have shown benefits as well [17–19]. The International Working Group and the United States National Institute on Aging--Alzheimer's Association working group proposed a series of diagnostic criteria for Alzheimer's disease to better define clinical phenotypes and integrate biomarkers into the diagnostic process [20–25]. However, because of the disease heterogeneity, inexact nature of imaging tests, and cohort differences, quantitative standardization of these ancillary tests needs more calibration [26–30]. Although a biomarkers classification scheme was proposed [31], behaviour tests still play an important role in the diagnostic process [32].

Recent development in machine learning provides opportunities to deal with the problem from a different aspect. Successful applications of deep convolutional neural network in imaging segmentation enabled accurate and automatic brain segmentation using machine learning

pipelines. Tools such as DeepNAT demonstrated accurate neuroanatomy segmentation [33,34]. Researchers also have developed end-to-end diagnosis pipelines for AD diagnosis [35,36]. However, one common critique is that these models are often hard to interpret [37]. Yet the high performance of these machine learning methods is enlightening, and an approach that could combine the predictive power and the interpretability would be exciting.

In this study, we propose a quantitative approach to address the issue of heterogeneity in the diagnostic process. Instead of modeling markers explicitly, we explored similarity-based diagnostic modeling on patient data---each incoming subject is compared to known individuals in the medical record database using a kernel function, and the Gaussian process method gives a diagnosis according to the similarity of medical conditions. Based on this idea, we developed a proof-of-concept Alzheimer's disease diagnostic model that uses a combination of demographic, genetic, and MRI data, but without behavioural test features. To evaluate the method, our model was benchmarked on ADNI dataset using cross-validation tests. It was also tested in the Alzheimer Disease Big Data DREAM Challenge on independent patient datasets and ranked highest among submitted methods [38,39]. To further explore the potential of the similarity-based modeling, we enhanced the model on predicting AD progression among patients in the mild-cognitive-impairment (MCI) group and extended the approach to the diagnosis of Parkinson's disease.

Methods

See **Supplementary information** for details [40–47].

Results

To address the problem of clinical heterogeneity, we developed a pipeline to match incoming subjects against known cases in the database and make diagnosis more similar to those of individuals with similar conditions. This strategy was formulated as a Gaussian process. A Gaussian process model utilizes a kernel function to measure the similarity of the known individuals in the databases [48], weights all cases proportionally to the inverse of their similarity to the incoming subjects, and reports a weighted mean of all known diagnoses. Thus, the diagnostic prediction is biased towards the diagnoses of subjects with similar conditions. Various test results can be incorporated into the kernel function. Instead of asking for thresholds or value ranges for these tests, the model learns the distribution of these test results implicitly. Importance of different tests can also be adjusted quantitatively. With a fitting kernel function, a Gaussian process model is able to circumvent the clinical heterogeneity issue and make diagnosis accurately.

As numerous reports have revealed a high degree of heterogeneity in Alzheimer's disease progression and clinical observations [29,49,50], we applied our approach to the diagnosis of Alzheimer's disease as a proof of concept. An overview of our complete diagnostic model is shown in Fig 1. For every subject, we collected demographic information, genetic data, and a MRI scan. MRI scans were preprocessed using FreeSurfer [43], ANT [44], and MindBoggle [45]. These programs labelled anatomic structures in the brain through image registration, performed

surface- and voxel-based morphometric analyses, and output statistics about sizes, surface areas, and cortical thickness of these anatomical structures. We chose a limited set of features from these data in our kernel function, including education levels, APOE allelic information, hippocampal volumes, amygdala volumes, and inferior lateral ventricle volumes. We also averaged the surface areas and volumes of multiple structures and incorporated them in to the kernel function. Hippocampal volumes are chosen because they are the most correlated features and have been described repeatedly in the literature. The remaining features are chosen based on forward feature selection through cross-validation tests. We then used the Gaussian Process Regression model to predict the cognitive impairment in terms of MMSE scores and to classify subjects into the cognitive normal (CN), mild cognitive impairment (MCI), and AD groups.

Formulating population similarity as a Gaussian process brought us a valuable tool to investigate disease heterogeneity. By mapping known individuals in the training dataset into a hyperspace, we investigated the correlation between the kernel-transformed feature similarity and the cognition similarity at the individual level. We also explored the extensibility of the similarity modeling approach: By incorporating subject-level similarity network analysis, we enhanced the model to identify MCI patients who were at high risks of MCI-to-AD conversion. To further validate the performance of our approach on heterogeneous neurological diseases in general, we also evaluated our algorithm on Parkinson's disease data.

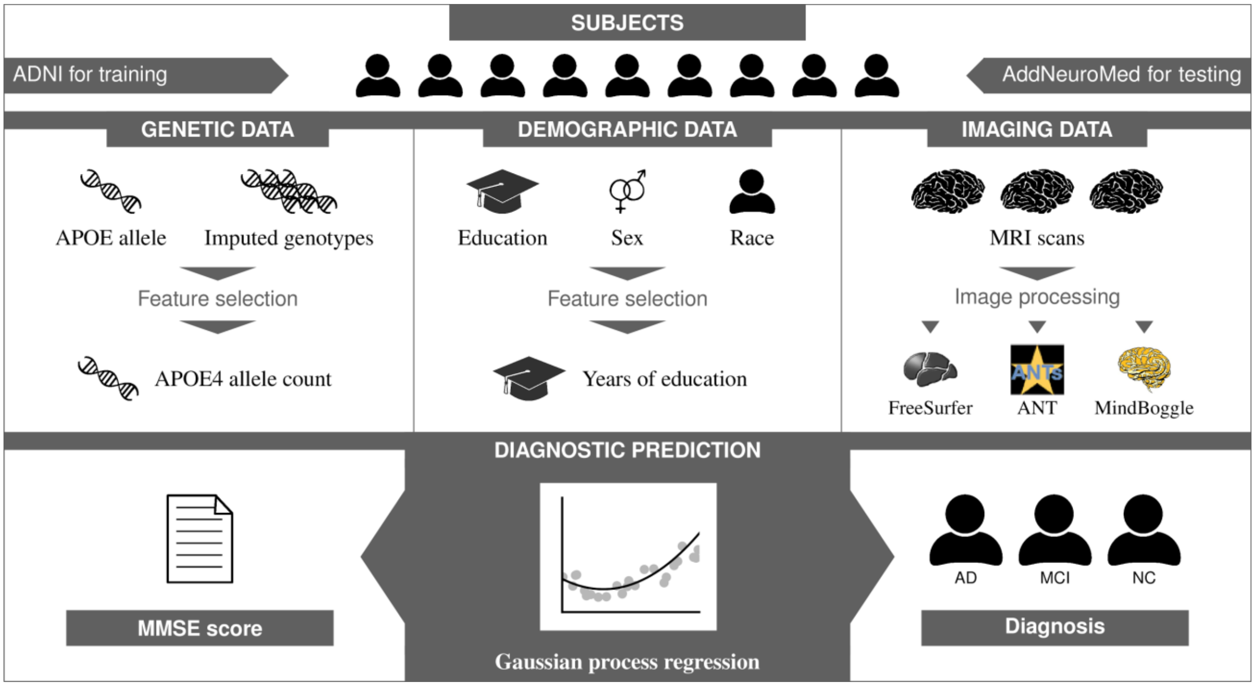

Figure 1. An overview of the Alzheimer’s disease diagnostic model.

Accurate prediction of cognitive impairment and diagnoses using similarity modeling

Alzheimer's disease was chosen as a case study because of its reportedly high degree of heterogeneity at various levels. As a benchmark test, we first evaluated our approach on Alzheimer's disease diagnosis and developed a model for estimating the severity of cognitive impairment and making a diagnosis using only baseline measurements without longitudinal information. We compared three major estimation strategies: linear modeling, decision tree modeling, and our similarity-based modeling. Linear models are well studied in many statistical models. They make strong assumptions about the linearity of factors. In this case, we tested linear regression models with different regularization schemes. Decision trees are similar to how human make decisions: they iteratively divide the samples based on highly discriminative factors, such as age groups or hippocampal volume ranges, and make predictions for subgroups. We tested random forest, gradient boosting regression tree, and XGBoost regreesor methods, which are widely used and have performed well in many machine learning studies. Similarity-based modeling corresponds to kernel methods in machine learning, where sample similarity is calculated using a devised distance function, and predictions are made accordingly. We tested support vector regressor (SVR) and Gaussian process regression model. All models were evaluated in a 5-time 5-fold cross-validation test. In a cross-validation test, the dataset was split into 5 parts. In each round, one part was withheld, and the models were trained with the remaining 4 parts. The performance of the models was then evaluated according to how well the models predicted on the withheld patient data. The test was repeated to reduce the variance of error estimation [47].

To show the performance of these three classes of diagnostic modeling, we benchmarked their performance in terms of cognition estimation and diagnosis accuracy on the ADNI1 dataset (**Fig 2**). We evaluated the accuracy of cognitive impairment in terms of Pearson correlation coefficient and Lin's concordance correlation coefficient. The two metrics focus on different aspects: A high Pearson correlation coefficient suggests that the prediction can be well aligned with the observed values linearly, while a high Lin's concordance correlation coefficient indicates the similarity between the intra-group distributions of two datasets [51]. In our tests, similarity-guided methods outperformed other methods by a large margin in both metrics. The Gaussian process method marginally outperformed the SVR model and became the best prediction model.

We also evaluated the diagnosis accuracy of all models. We tested making a diagnosis based on the cognitive impairment predictions and evaluated the performance in terms of area under curve (AUC) of normal against AD/MCI classification. The Gaussian process method again outperformed other methods. We then further optimized the parameters of the model (**Supplementary Figure 1**). The final model was also tested on a hidden dataset from AddNeuroMed in the DREAM Challenge and predicted the cognition scores with a Pearson correlation coefficient of 0.573. Its performance on an independent multi-sectional study was consistently good in comparison to its performance in the cross-validation tests. We then moved on to study individual similarity estimation, subtype identification, and extensibility of the optimized similarity model.

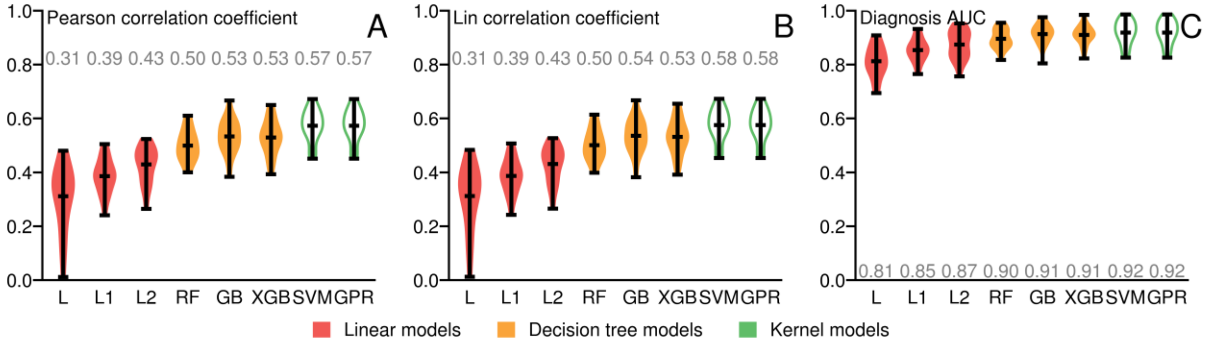

Figure 2. Violin plots of performance of different models estimated by cross-validation. (A) Performance of MMSE regression evaluated in terms of Pearson product-moment correlation coefficient. (B) Performance of MMSE regression evaluated in terms of Lin's concordance correlation coefficient. (C) Performance of diagnostic predictions evaluated in terms of AUC. The average scores are labelled correspondingly. The final model performance is marked with a white body. (GPR=Gaussian process regression with custom kernel, SVR=kernel support vector regressor, GB=gradient boosting regression tree, RF=random forest, XGB=XGBoost, L1=LASSO regression, L2=ridge regression, L=linear regression without regularisation).

Subject-level analysis on the severity of cognitive impairment using kernel methods

Working with heterogeneous data from multi-sectional study poses a challenge to data modeling. We addressed the heterogeneity issue by matching prediction targets to subjects with similar conditions using a kernel function and thus avoided specifying explicit thresholds or ranges for biomarkers across different cohorts. Since the similarity model is the core of our strategy, we assessed the effectiveness of our similarity model on the ADNI1 dataset. We performed Principal Component Analysis (PCA) over two different similarity matrices, one calculated from a dot-product similarity (which is equivalent to linear regression model) and the other from our custom kernel. With the custom kernel, PCA showed a strong correlation between Alzheimer disease progression and transformed MRI features (**Fig 3C**). It also clustered samples according to risk factors such as APOE ε4 allelic count (**Fig 3D**). Such a pattern was not clearly observed from the dot-product similarity model (**Fig 3A--B**). Visualization of the spatial distribution of samples before and after kernel transformation suggested that the kernel might extract a strong signal and estimate individual similarity well.

To validate our hypothesis from the above analysis, we then compared the results of the similarity functions to the similarity of cognition of individuals. For each sample, we calculated its feature-wise similarity to all other samples using both dot-product and custom kernel functions and then computed dissimilarity correlation scores (DCSs). DCSs quantify the correlation between cognitive impairment differences and the reciprocals of their similarity (**Supplementary Figure 2**). An effective similarity measurement is expected to have a high DCS, showing that feature-wise dissimilarity correlates with diagnostic dissimilarity. The kernel transformation was significantly better than random permutation (one sided t-test $p<<0.001$) and significantly improved DCS over the linear similarity model (one-sided t-test $p<<0.001$). Both visualization

and quantitative analysis suggested that the kernel methods gave good estimation on cognition similarity between individuals with various clinical features.

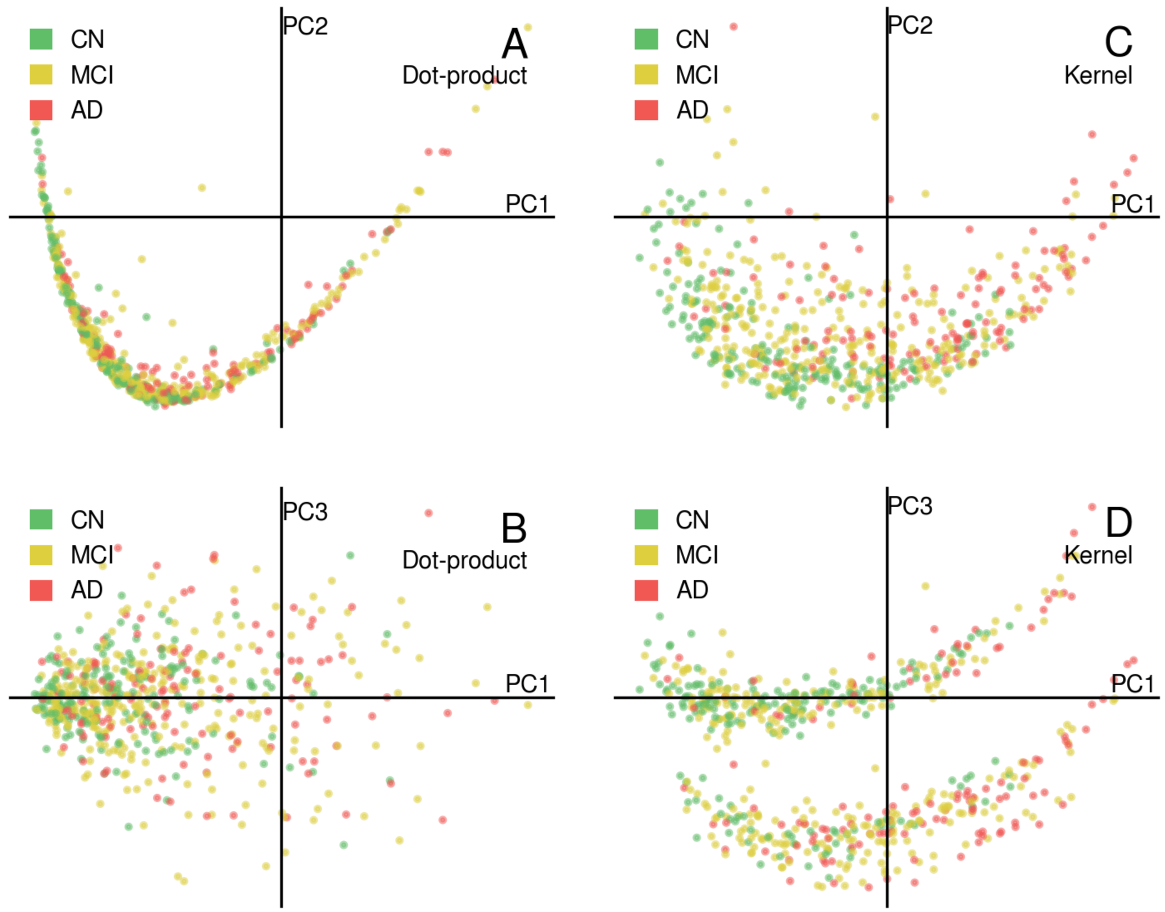

Figure 3. Principal component analysis (PCA) over the dot-product similarity matrix (A & B) and the custom kernel similarity matrix (C & D). PCA on kernel matrix revealed patterns of different disease progressions in the transformed feature space.

Applications to Alzheimer's disease progression and Parkinson's disease diagnostic predictions

Beyond estimating the cognitive impairment and predicting AD diagnoses, our model can be extended to other scenarios. For example, an important aspect in AD study is identifying MCI patients that might soon convert to AD. Here, we extended our diagnostic model to a progression prediction model with only baseline measurements by incorporating network analysis. To investigate the specificity of our approach in regards to these two vulnerable subgroups, we first looked into the predictions of our diagnostic model. (See Supplementary Table 2 for the confusion matrix of our prediction evaluation.) While our approach achieved a high AUC in diagnosing MCI/AD subjects against normal subjects, 110 out of 296 MCI patients

were misclassified as AD patients. In this case, our diagnostic model made predictions based on patient similarity and considered these patients to be more similar to AD patients than other MCI patients. Thus, we compared this result to the longitudinal data present in the ADNI database. Of 110 MCI subjects who were predicted as AD patients, 75 (68%) eventually converted into AD in the later follow-up examinations during 4 years. The AD predictions were significantly biased towards patients who later converted to AD (Fisher exact test $p \ll 0.001$).

The uneven distribution" (of misclassification cases), "provided that our algorithm predicts diagnosis based on patient similarity, suggests the correlation between the similarity of these MCI patients to AD and normal subjects and their disease progression. More specifically, MCI patients whose conditions resemble those of AD patients might face higher risks of AD conversion, while other MCI patients might be less likely to develop AD. To test the hypothesis, we analyzed the patient similarity network. We built a network that connects subjects in the training dataset with edges. The weights of the edges are the similarity between connecting subjects calculated by the kernel function in our algorithm. Under our hypothesis, highly weighted edges would associate MCI patients that converted later to AD patients more closely than normal subjects in the network. To analyze the connective patterns of highly weighted edges, we first trimmed the network by filtering out the lowest 97.5% weighted edges and then applied Girvan-Newman community clustering algorithm (as implemented in clusterMaker2, a Cytoscape plugin). We chose the threshold by comparing the modularity of the final clustering results against that of trimmed and clustered random networks, and the threshold level of 97.5% achieved the most significant difference. Girvan-Newman clustering algorithm decomposed the trimmed network into 10 clusters (**Figure 4**). We dropped the smallest 4 clusters out of the analysis, each of which has less than 10 subjects. Among the remaining clusters, Cluster 1, 3, and 6 contained more normal subjects than AD patients, while Cluster 2, 4, and 5 contains more AD patients. Based on the clustering results, we directly predict that those MCI patients in Cluster 1, 3, and 6 have low risks in disease progression, as well as those in the other clusters have high risks. A Fisher exact test on these subjects confirmed the discriminative power ($p = 0.0001$). Despite not including any longitudinal data, our model successfully captured the properties of the subpopulation which is vulnerable to MCI-to-AD conversion. It demonstrated the effectiveness of the similarity function we adopted in the prediction model.

The idea of using subjects with similar conditions to guide diagnosis is not specific to AD, and the model we proposed in this study can be applied to other diseases as well. To demonstrate its extensibility, we built a Gaussian process diagnosis model for Parkinson's disease. The model was trained on the Parkinson's Progression Markers Initiative dataset (Marek et al., 2011). The MRI images were processed using FreeSurfer to extract numeric features such as surface areas and volumes. We included ages and genders as features, together with areas and volumes of brain anatomic structures estimated by FreeSurfer. We performed repeated 5-fold cross validation on 302 subjects in the dataset. The Gaussian process model achieved an AUC of ~0.88 (**Figure 5**, **Supplementary Figure 3**). Thus, for heterogeneous neurological diseases other than AD, the similarity-based diagnostic strategy can give accurate diagnostic predictions as well.

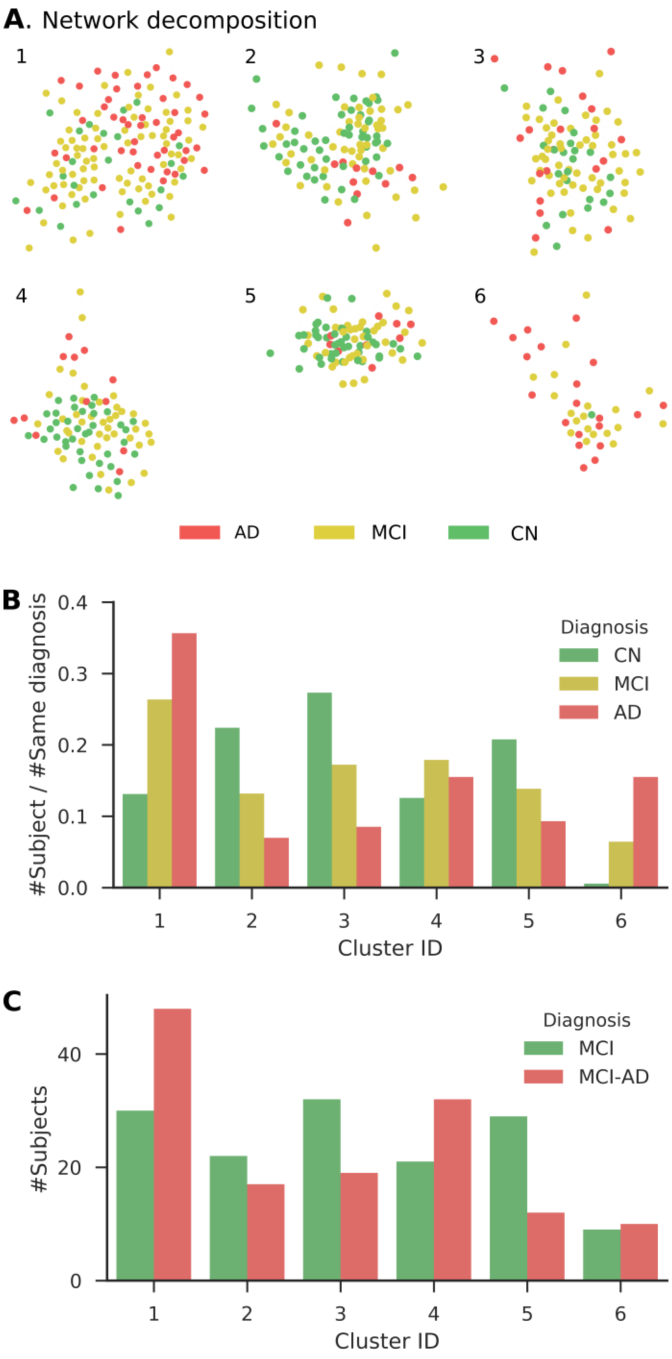

Figure 4. Similarity networks of subjects in ADNI dataset, decomposited by Girvan-Newman algorithm. (A) Visualization of the clusters. Subjects are colored according to baseline diagnosis. (B) The distribution of three diagnostic types in two clusters. (C) The distribution of MCI-to-AD conversion/non-conversion subjects in two clusters.

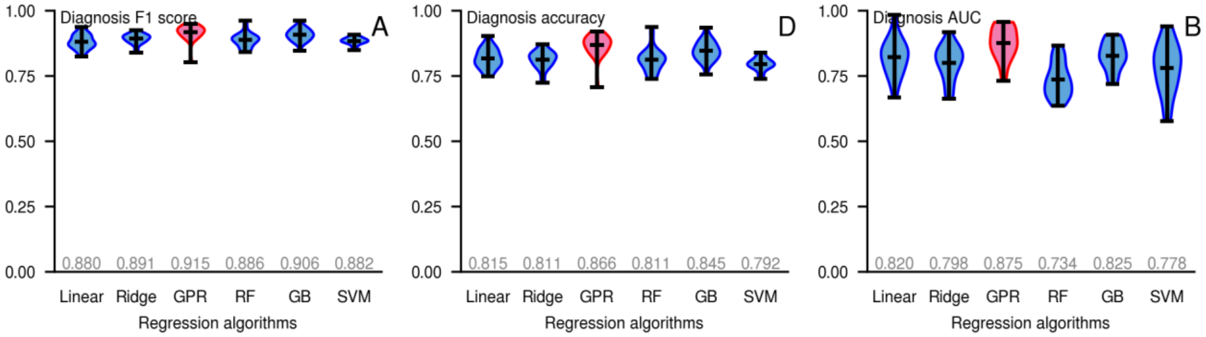

Figure 5. Violin plots of performance of different diagnostic models estimated on Parkinson's disease dataset by cross-validation. Performance is evaluted in terms of F1 scores, ratio of correct predictions, and AUC.The average scores are labelled correspondingly. (GPR=Gaussian process regression with custom kernel, SVR=kernel support vector regressor, GB=gradient boosting regression tree, RF=random forest, Ridge=ridge regression, Linear=linear regression without regularisation).

Discussions

In this study, we show that similarity-based diagnostic modeling is an effective approach to deal with heterogeneous diseases and non-linear clinical data. The modeling focuses on subjects with similar conditions, and uses their diagnoses to guide our decisions. We tested the method in Alzheimer's disease and achieved very good performance in estimating the severity of cognitive impairment indicated by MMSE scores and predicting diagnoses. Specifically for MCI patients, the model even captured the differences between those who would later convert to AD later and those who would not. We also tested the method in Parkinson's disease to show the generality of the modeling approach.

The idea of similarity modeling applied here to disease diagnosis echoes recent studies on population modeling. Various social network studies demonstrated that local similarity predicts various properties of individuals [52–54]. Adapting this idea from social network settings, previous studies found that the similarity approach can be used to estimate disease risks [55–57]. Here, we focused on individual diseases where heterogeneity hinders accurate diagnosis. Previously we showed that a Gaussian process is effective in handling medical datasets of limited samples [58,59], and recent reports indicate that it can also be extended to larger scale studies [60].

While the idea of similarity modeling has been applied to many topics, our method still needed to solve four major challenges specific to diagnostic modeling: it must deal with heterogeneous nonlinear data, remain interpretable in medical contexts, provide insights into disease progression, and be effective on different cohorts, new assays, and even other diseases. A method that achieved these goals would bridge the gap between conventional diagnostic models and advanced machine learning models in terms of predictive power, interpretability, perceptiveness, and flexibility. We paid close attention to these four focuses throughout the study:

First, our similarity approach is a non-parametric approach that does not assume the underlying distributions of risk factors [61]. Commonly adopted methods often assume independence and linear relationship between features for simplicity and work adequately for some cases; yet for diseases that manifest heterogeneous phenotypes, such an assumption can be incorrect and a more complicated model may be necessary [62]. This is particularly true when dealing with multi-cohort datasets, where batch effects can further obscure the relationship between observed measurements and clinical outcomes [63]. In comparison to many commonly used regression methods, our nonparametric approach is more flexible in dealing with nonlinear data. The kernel function brings more accurate modeling and stronger predictive power.

Second, our approach is a well-studied statistical model which, unlike many other advanced machine learning models, can be easily interpreted. Many advanced machine learning models have been developed for diagnosis of Alzheimer's disease [64], especially various deep neural network models [36,65–67]. While most of these models show great performance in diagnostic classification, interpreting deep learning models remains a hard problem [68]. Uninterpretable models in medical applications can be undesirable in some cases [37,69]. A Gaussian process, on the other hand, is a well-studied statistical model and takes an intuitive approach. The prediction process can be performed with little human intervention, and yet physicians can still read the estimated confidence interval from Gaussian process regression to judge how confident the diagnosis is. The similarity of individual subjects reported from the model also tells the reason behind the prediction. A bonus point to our approach is that it is possible to combine the interpretability of Gaussian process and the power of deep neural network together through techniques such as ensemble model and stack generalization [70].

Third, our method identifies subjects that manifest different disease progression rates through the pattern of the similarity network. Most excitingly, we found that our model accomplished this when fed only baseline information and lacked any longitudinal information of training subjects. Previous research showed that MCI patients who would later convert to AD show similar traits to AD patients in their baseline measurements [71,72]. It allows our model to capture features to predict disease progression. The network provides a different view of the subjects and can be a powerful tool for researchers to investigate the heterogeneity of disease progression at a population level [57,73]. Diagnosis of other similar diseases which show heterogeneity in disease progression, could benefit from the application of our network analysis approach.

Finally, our method can be easily transferred to different cohorts, assays, or diseases. In the Alzheimer Disease Big Data DREAM Challenge, our model has been tested on AddNeuroMed, an independent multi-cohort Alzheimer's disease study focusing on European subjects [42], and achieved consistently high performance [38]. The feature input to our method allows incorporation of imaging data, genotype information, biochemical marker assays, and many other tests. Recent studies on Alzheimer's disease risk factors can be incorporated into the similarity function [11,74,75]. Here we have shown its application to the diagnosis of Parkinson's disease, and previously we developed Gaussian process models for other diseases [58]. The model demonstrates extensibility and flexibility in dealing with different datasets without compromising its state-of-the-art predictive power in diagnostic prediction.

While our approach dealt with the four challenges listed above, there is still room for improvement. While our Alzheimer's disease model achieved promising results, it was

developed under rather harsh settings (required by the challenge for competition fairness). The DREAM Challenge chose MMSE as one of the prediction targets of the competition, yet studies have shown MMSE is not an indicator specific enough for Alzheimer's disease [76,77]; for the challenge, no other disease-specific indicators were available. In addition, the MRI images were automatically processed by programs without manual intervention, and recent studies suggest the accuracy of this auto-labelling pipeline can be further improved [78,79], which would boost the performance. Fortunately, these limitations can be avoided or solved in real world application, in which case our method would achieve even better results. Further applications to different cohorts, clinical tests, or diseases will be of interest. On the computational side, kernel matrix calculation requires comparing all pairs of samples. The time and memory complexity grows quadratically when the number of samples increases. Researchers have found approximation of kernel calculations for large datasets, but their effects on prediction accuracy of our model needs further investigation.

Conclusions

We presented a novel computational approach that estimates the cognitive impairment of Alzheimer's patients. The method calculates the similarity between subjects using structural MRI data and other clinical measurements from multi-sectional studies, and predicts cognitive impairment with biases towards patients with high similarity. The method, fed with ADNI1 data, demonstrated its state-of-the-art predictive power on disease diagnosis. The idea of relating incoming subjects to known cases of similar conditions allows more specialized diagnosis; without any information about disease progression, unsupervised similarity network analysis predicted patients at high risks of MCI-to-AD conversion. The effectiveness of this similarity-based approach was validated on an independent cohort and tested on Parkinson's disease, another common, heterogeneous neurological disease. The promising performance of our model suggests that not only can it be an alternative approach to establishing quantitative diagnostic criteria, it also represents an attractive tool for researchers to study disease progression.

Acknowledgements

This work was supported by the National Institutes of Health and National Institute of Aging [P30AG053760 to Michigan Alzheimer Disease Core Center], National Science Foundation [1452656], and Alzheimer's Association [Cross-disease brain image modeling].

We appreciate Dr. Henry Paulson for valuable suggestions.

Data collection and sharing for this project was funded by the Alzheimer's Disease Neuroimaging Initiative (ADNI) (National Institutes of Health Grant U01 AG024904) and DOD ADNI (Department of Defense award number W81XWH-12-2-0012). ADNI is funded by the National Institute on Aging, the National Institute of Biomedical Imaging and Bioengineering, and through generous contributions from the following: AbbVie; Alzheimer’s Association; Alzheimer’s Drug Discovery Foundation; Araclon Biotech; BioClinica, Inc.; Biogen; Bristol-Myers Squibb Company; CereSpir, Inc.; Cogstate; Eisai Inc.; Elan Pharmaceuticals, Inc.; Eli Lilly and

Company; EuroImmun; F. Hoffmann-La Roche Ltd and its affiliated company Genentech, Inc.; Fujirebio; GE Healthcare; IXICO Ltd.; Janssen Alzheimer Immunotherapy Research & Development, LLC.; Johnson & Johnson Pharmaceutical Research & Development LLC.; Lumosity; Lundbeck; Merck & Co., Inc.; Meso Scale Diagnostics, LLC.; NeuroRx Research; Neurotrack Technologies; Novartis Pharmaceuticals Corporation; Pfizer Inc.; Piramal Imaging; Servier; Takeda Pharmaceutical Company; and Transition Therapeutics. The Canadian Institutes of Health Research is providing funds to support ADNI clinical sites in Canada. Private sector contributions are facilitated by the Foundation for the National Institutes of Health (www.fnih.org). The grantee organization is the Northern California Institute for Research and Education, and the study is coordinated by the Alzheimer’s Therapeutic Research Institute at the University of Southern California. ADNI data are disseminated by the Laboratory for Neuro Imaging at the University of Southern California.

PPMI---a public-private partnership---is funded by the Michael J. Fox Foundation for Parkinson’s Research and funding partners, including Abbvie; Avid Radiopharmaceutical; Biogen; Biolegend; Bristol-Myers Squibb Company; GE Healthcare; Genentech; GlaxoSmithKline; Golub Capital; Eli Lilly & Co.; Lundbeck; Merck & Co., Inc.; Meso Scale Discovery; Pfizer Inc.; Piramal Imaging; Roche; Sanofi Genzyme; Servier; Takeda Pharmaceutical Company; Teva; UCB.

Competing interests

The authors declare that they have no competing interests.

Availability of supporting data and materials

The original competition code, together with demonstration data, is available at https://www.synapse.org/#!Synapse:syn2527678/wiki/69937 .Additional code is available at https://github.com/GuanLab/2014_AD_Sup. Data is also available in the *GigaScience* GigaDB repository[80].

Abbreviations

AD: Alzheimer's Disease ADNI: Alzheimer's Disease Neuroimaging Initiative; AUC: area under curve; CN: cognitive normal; DCS: dissimilarity correlation scores; MCI: mild-cognitive-impairment; MRI: Magnetic resonance imaging; PCA: Principal Component Analysis; SVR: support vector regressor.

1. Scott KR, Barrett AM. Dementia syndromes: evaluation and treatment. Expert Rev. Neurother. 2007;7:407–22.

2. Banerjee S, Wittenberg R. Clinical and cost effectiveness of services for early diagnosis and intervention in dementia. Int. J. Geriatr. Psychiatry. 2009;24:748–54.

3. 2017 Alzheimer’s disease facts and figures. Alzheimers. Dement. 2017;13:325–73.

4. Murray CJL, Vos T, Lozano R, Naghavi M, Flaxman AD, Michaud C, et al. Disability-adjusted life years (DALYs) for 291 diseases and injuries in 21 regions, 1990-2010: a systematic analysis for the Global Burden of Disease Study 2010. Lancet. 2012;380:2197–223.

5. Thakur KT, Albanese E, Giannakopoulos P, Jette N, Linde M, Prince MJ, et al. Neurological Disorders. The International Bank for Reconstruction and Development / The World Bank; 2016 [cited 2017 Oct 20]; Available from: https://www.ncbi.nlm.nih.gov/books/NBK361950/

6. Buckley JS, Salpeter SR. A Risk-Benefit Assessment of Dementia Medications: Systematic Review of the Evidence. Drugs Aging. 2015;32:453–67.

7. Yiannopoulou KG, Papageorgiou SG. Current and future treatments for Alzheimer’s disease. Ther. Adv. Neurol. Disord. 2012;6:19–33.

8. Galvin JE, Sadowsky CH, NINCDS-ADRDA. Practical guidelines for the recognition and diagnosis of dementia. J. Am. Board Fam. Med. 2012;25:367–82.

9. Agrawal M, Biswas A. Molecular diagnostics of neurodegenerative disorders. Front Mol Biosci. 2015;2:54.

10. Blennow K. Cerebrospinal fluid protein biomarkers for Alzheimer’s disease. NeuroRx. 2004;1:213–25.

11. Snowden SG, Ebshiana AA, Hye A, An Y, Pletnikova O, O’Brien R, et al. Association between fatty acid metabolism in the brain and Alzheimer disease neuropathology and cognitive performance: A nontargeted metabolomic study. PLoS Med. 2017;14:e1002266.

12. Frisoni GB, Fox NC, Jack CR Jr, Scheltens P, Thompson PM. The clinical use of structural MRI in Alzheimer disease. Nat. Rev. Neurol. 2010;6:67–77.

13. Vemuri P, Wiste HJ, Weigand SD, Shaw LM, Trojanowski JQ, Weiner MW, et al. MRI and CSF biomarkers in normal, MCI, and AD subjects: Diagnostic discrimination and cognitive correlations. Neurology. 2009;73:287–93.

14. Vemuri P, Wiste HJ, Weigand SD, Shaw LM, Trojanowski JQ, Weiner MW, et al. MRI and CSF biomarkers in normal, MCI, and AD subjects: Predicting future clinical change. Neurology. 2009;73:294–301.

15. Thompson PM, Hayashi KM, De Zubicaray GI, Janke AL, Rose SE, Semple J, et al. Mapping hippocampal and ventricular change in Alzheimer disease. Neuroimage. 2004;22:1754–66.

16. Thompson PM, Hayashi KM, de Zubicaray G, Janke AL, Rose SE, Semple J, et al. Dynamics of gray matter loss in Alzheimer’s disease. J. Neurosci. 2003;23:994–1005.

17. Sabri O, Sabbagh MN, Seibyl J, Barthel H, Akatsu H, Ouchi Y, et al. Florbetaben PET imaging to detect amyloid beta plaques in Alzheimer’s disease: phase 3 study. Alzheimers. Dement. 2015;11:964–74.

18. Zhang W, Arteaga J, Cashion DK, Chen G, Gangadharmath U, Gomez LF, et al. A highly selective and specific PET tracer for imaging of tau pathologies. J. Alzheimers. Dis.

2012;31:601–12.

19. Clark CM, Schneider JA, Bedell BJ, Beach TG, Bilker WB, Mintun MA, et al. Use of florbetapir-PET for imaging beta-amyloid pathology. JAMA. 2011;305:275–83.

20. McKhann GM, Knopman DS, Chertkow H, Hyman BT, Jack CR Jr, Kawas CH, et al. The diagnosis of dementia due to Alzheimer's disease: recommendations from the National Institute on Aging-Alzheimer's Association workgroups on diagnostic guidelines for Alzheimer's disease. Alzheimers. Dement. 2011;7:263–9.

21. Albert MS, DeKosky ST, Dickson D, Dubois B, Feldman HH, Fox NC, et al. The diagnosis of mild cognitive impairment due to Alzheimer's disease: Recommendations from the National Institute on Aging-Alzheimer's Association workgroups on diagnostic guidelines for Alzheimer's disease. Alzheimers. Dement. 2011;7:270–9.

22. Sperling RA, Aisen PS, Beckett LA, Bennett DA, Craft S, Fagan AM, et al. Toward defining the preclinical stages of Alzheimer's disease: recommendations from the National Institute on Aging-Alzheimer's Association workgroups on diagnostic guidelines for Alzheimer's disease. Alzheimers. Dement. 2011;7:280–92.

23. Jack CR, Albert MS, Knopman DS, McKhann GM, Sperling RA, Carrillo MC, et al. Introduction to the recommendations from the National Institute on Aging-Alzheimer's Association workgroups on diagnostic guidelines for Alzheimer's disease. Alzheimers. Dement. 2011;7:257–62.

24. Dubois B, Feldman HH, Jacova C, Cummings JL, Dekosky ST, Barberger-Gateau P, et al. Revising the definition of Alzheimer's disease: a new lexicon. Lancet Neurol. 2010;9:1118–27.

25. Dubois B, Feldman HH, Jacova C, Dekosky ST, Barberger-Gateau P, Cummings J, et al. Research criteria for the diagnosis of Alzheimer's disease: revising the NINCDS-ADRDA criteria. Lancet Neurol. 2007;6:734–46.

26. Klöppel S, Abdulkadir A, Jack CR, Koutsouleris N, Mourão-Miranda J, Vemuri P. Diagnostic neuroimaging across diseases. Neuroimage. 2012;61:457–63.

27. McEvoy LK, Brewer JB. Quantitative structural MRI for early detection of Alzheimer's disease. Expert Rev. Neurother. 2010;10:1675–88.

28. Wollman DE, Prohovnik I. Sensitivity and specificity of neuroimaging for the diagnosis of Alzheimer's disease. Dialogues Clin. Neurosci. 2003;5:89–99.

29. Lam B, Masellis M, Freedman M, Stuss DT, Black SE. Clinical, imaging, and pathological heterogeneity of the Alzheimer's disease syndrome. Alzheimers. Res. Ther. 2013;5:1.

30. Thompson WK, Hallmayer J, O'Hara R, Alzheimer's Disease Neuroimaging Initiative. Design considerations for characterizing psychiatric trajectories across the lifespan: application to effects of APOE-ε4 on cerebral cortical thickness in Alzheimer's disease. Am. J. Psychiatry. 2011;168:894–903.

31. Jack CR, Bennett DA, Blennow K, Carrillo MC, Feldman HH, Frisoni GB, et al. A/T/N: An unbiased descriptive classification scheme for Alzheimer disease biomarkers. Neurology. 2016;87:539–47.

32. Kennedy RE, Cutter GR, Wang G, Schneider LS. Using baseline cognitive severity for enriching Alzheimer's disease clinical trials: How does Mini-Mental State Examination predict rate of change? Alzheimers. Dement. 2015;1:46–52.

33. Wachinger C, Reuter M, Klein T. DeepNAT: Deep convolutional neural network for segmenting neuroanatomy. Neuroimage. 2017; http://dx.doi.org/10.1016/j.neuroimage.2017.02.035

34. de Brebisson A, Montana G. Deep neural networks for anatomical brain segmentation. 2015 IEEE Conference on Computer Vision and Pattern Recognition Workshops (CVPRW) [Internet]. 2015. Available from: http://dx.doi.org/10.1109/cvprw.2015.7301312

35. Sarraf S, Tofighi G. Deep learning-based pipeline to recognize Alzheimer's disease using fMRI data. 2016 Future Technologies Conference (FTC) [Internet]. 2016. Available from: http://dx.doi.org/10.1109/ftc.2016.7821697

36. Suk H-I, Shen D. Deep Learning-Based Feature Representation for AD/MCI Classification. Lecture Notes in Computer Science. 2013. p. 583–90.

37. Caruana R, Lou Y, Gehrke J, Koch P, Sturm M, Elhadad N. Intelligible Models for HealthCare. Proceedings of the 21th ACM SIGKDD International Conference on Knowledge Discovery and Data Mining - KDD '15 [Internet]. 2015. Available from: http://dx.doi.org/10.1145/2783258.2788613

38. Allen GI, Amoroso N, Anghel C, Balagurusamy V, Bare CJ, Beaton D, et al. Crowdsourced estimation of cognitive decline and resilience in Alzheimer's disease. Alzheimers. Dement. 2016;12:645–53.

39. Zhu F, Panwar B, Dodge HH, Li H, Hampstead BM, Albin RL, et al. COMPASS: A computational model to predict changes in MMSE scores 24-months after initial assessment of Alzheimer's disease. Sci. Rep. [Internet]. 2016;6. Available from: http://dx.doi.org/10.1038/srep34567

40. Mueller SG, Weiner MW, Thal LJ, Petersen RC, Jack C, Jagust W, et al. The Alzheimer's disease neuroimaging initiative. Neuroimaging Clin. N. Am. 2005;15:869–77, xi – xii.

41. Jack CR Jr, Bernstein MA, Fox NC, Thompson P, Alexander G, Harvey D, et al. The Alzheimer's Disease Neuroimaging Initiative (ADNI): MRI methods. J. Magn. Reson. Imaging. 2008;27:685–91.

42. Lovestone S, Francis P, Kloszewska I, Mecocci P, Simmons A, Soininen H, et al. AddNeuroMed--the European collaboration for the discovery of novel biomarkers for Alzheimer's disease. Ann. N. Y. Acad. Sci. 2009;1180:36–46.

43. Fischl B. FreeSurfer. Neuroimage. 2012;62:774–81.

44. Tustison NJ, Cook PA, Klein A, Song G, Das SR, Duda JT, et al. Large-scale evaluation of ANTs and FreeSurfer cortical thickness measurements. Neuroimage. 2014;99:166–79.

45. Klein A, Hirsch J. Mindboggle: a scatterbrained approach to automate brain labeling. Neuroimage. 2005;24:261–80.

46. Parkinson Progression Marker Initiative. The Parkinson Progression Marker Initiative

(PPMI). Prog. Neurobiol. 2011;95:629–35.

47. Kim J-H. Estimating classification error rate: Repeated cross-validation, repeated hold-out and bootstrap. Comput. Stat. Data Anal. 2009;53:3735–45.

48. Schölkopf B, Tsuda K, Vert J-P. Kernel Methods in Computational Biology. MIT Press; 2004.

49. Komarova NL, Thalhauser CJ. High Degree of Heterogeneity in Alzheimer's Disease Progression Patterns. PLoS Comput. Biol. 2011;7:e1002251.

50. Dodge HH, Zhu J, Harvey D, Saito N, Silbert LC, Kaye JA, et al. Biomarker progressions explain higher variability in stage-specific cognitive decline than baseline values in Alzheimer disease. Alzheimers. Dement. 2014;10:690–703.

51. Nickerson CAE. A Note On "A Concordance Correlation Coefficient to Evaluate Reproducibility." Biometrics. 1997;53:1503.

52. Liu C, Liu J, Jiang Z. A multiobjective evolutionary algorithm based on similarity for community detection from signed social networks. IEEE Trans Cybern. 2014;44:2274–87.

53. Otte E. Social network analysis: a powerful strategy, also for the information sciences. J. Inf. Sci. Eng. 2002;28:441–54.

54. Dodds PS, Harris KD, Kloumann IM, Bliss CA, Danforth CM. Temporal Patterns of Happiness and Information in a Global Social Network: Hedonometrics and Twitter. PLoS One. 2011;6:e26752.

55. Sharafoddini A, Dubin JA, Lee J. Patient Similarity in Prediction Models Based on Health Data: A Scoping Review. JMIR Med Inform. 2017;5:e7.

56. Ng K, Sun J, Hu J, Wang F. Personalized Predictive Modeling and Risk Factor Identification using Patient Similarity. AMIA Jt Summits Transl Sci Proc. 2015;2015:132–6.

57. Li L, Cheng W-Y, Glicksberg BS, Gottesman O, Tamler R, Chen R, et al. Identification of type 2 diabetes subgroups through topological analysis of patient similarity. Sci. Transl. Med. 2015;7:311ra174–311ra174.

58. Sieberts SK, Zhu F, García-García J, Stahl E, Pratap A, Pandey G, et al. Crowdsourced assessment of common genetic contribution to predicting anti-TNF treatment response in rheumatoid arthritis. Nat. Commun. 2016;7:12460.

59. Varol E, Gaonkar B, Erus G, Schultz R, Davatzikos C. Feature ranking based nested support vector machine ensemble for medical image classification. 2012 9th IEEE International Symposium on Biomedical Imaging (ISBI) [Internet]. 2012. Available from: http://dx.doi.org/10.1109/isbi.2012.6235505

60. Wilson AG, Dann C, Nickisch H. Thoughts on Massively Scalable Gaussian Processes [Internet]. 2015 [cited 2017 Oct 20]. Available from: http://arxiv.org/abs/1511.01870

61. Rasmussen CE, Williams CKI. Gaussian Processes for Machine Learning. Mit Press; 2006.

62. Mungas D, Beckett L, Harvey D, Farias ST, Reed B, Carmichael O, et al. Heterogeneity of cognitive trajectories in diverse older persons. Psychol. Aging. 2010;25:606–19.

63. Chen T, Chen L. Prediction of Clinical Outcome for All Stages and Multiple Cell Types of Non-small Cell Lung Cancer in Five Countries Using Lung Cancer Prognostic Index. EBioMedicine. 2014;1:156–66.

64. Mirzaei G, Adeli A, Adeli H. Imaging and machine learning techniques for diagnosis of Alzheimer's disease. Rev. Neurosci. [Internet]. 2016;27. Available from: http://dx.doi.org/10.1515/revneuro-2016-0029

65. Shen D, Wu G, Suk H-I. Deep Learning in Medical Image Analysis. Annu. Rev. Biomed. Eng. NIH Public Access; 2017;19:221.

66. Ortiz A, Munilla J, Górriz JM, Ramírez J. Ensembles of Deep Learning Architectures for the Early Diagnosis of the Alzheimer's Disease. Int. J. Neural Syst. 2016;26:1650025.

67. Li R, Zhang W, Suk H-I, Wang L, Li J, Shen D, et al. Deep learning based imaging data completion for improved brain disease diagnosis. Med. Image Comput. Comput. Assist. Interv. 2014;17:305–12.

68. Denker J, Schwartz D, Wittner B, Solla S, Howard R, Jackel L, et al. Large automatic learning, rule extraction, and generalization. Complex Systems.1987; 1: 877-922.

69. Che Z, Purushotham S, Khemani R, Liu Y. Interpretable Deep Models for ICU Outcome Prediction. AMIA Annu. Symp. Proc. 2016;2016:371–80.

70. Wolpert DH. Stacked generalization. Neural Netw. 1992;5:241–59.

71. Risacher SL, Saykin AJ, West JD, Shen L, Firpi HA, McDonald BC, et al. Baseline MRI predictors of conversion from MCI to probable AD in the ADNI cohort. Curr. Alzheimer Res. 2009;6:347–61.

72. Davatzikos C, Bhatt P, Shaw LM, Batmanghelich KN, Trojanowski JQ. Prediction of MCI to AD conversion, via MRI, CSF biomarkers, and pattern classification. Neurobiol. Aging. 2011;32:2322.e19–27.

73. Brown S-A. Patient Similarity: Emerging Concepts in Systems and Precision Medicine. Front. Physiol. 2016;7:561.

74. Lanoiselée H-M, Nicolas G, Wallon D, Rovelet-Lecrux A, Lacour M, Rousseau S, et al. APP, PSEN1, and PSEN2 mutations in early-onset Alzheimer disease: A genetic screening study of familial and sporadic cases. PLoS Med. 2017;14:e1002270.

75. Desikan RS, Fan CC, Wang Y, Schork AJ, Cabral HJ, Cupples LA, et al. Genetic assessment of age-associated Alzheimer disease risk: Development and validation of a polygenic hazard score. PLoS Med. 2017;14:e1002258.

76. Chapman KR, Bing-Canar H, Alosco ML, Steinberg EG, Martin B, Chaisson C, et al. Mini Mental State Examination and Logical Memory scores for entry into Alzheimer's disease trials. Alzheimers. Res. Ther.. 2016;8. http://dx.doi.org/10.1186/s13195-016-0176-z

77. Pengas G, Williams GB, Acosta-Cabronero J, Ash TWJ, Hong YT, Izquierdo-Garcia D, et al. The relationship of topographical memory performance to regional neurodegeneration in Alzheimer's disease. Front. Aging Neurosci. 2012;4:17.

78. Huo Y, Plassard AJ, Carass A, Resnick SM, Pham DL, Prince JL, et al. Consistent cortical reconstruction and multi-atlas brain segmentation. Neuroimage. 2016;138:197–210.

79. Choi H, Jin KH. Fast and robust segmentation of the striatum using deep convolutional neural networks. J. Neurosci. Methods. 2016;274:146–53.

80. Zhang, H; Zhu, F; Dodge, H, H; Higgins, G, A; Omenn, G, S; Guan, Y (2018): Supporting data for "A similarity-based approach to leverage multi-cohort medical data on the diagnosis and prognosis of Alzheimer's disease" GigaScience Database. http://dx.doi.org/10.5524/100471

Figure 1

Click here to access/download;Figure;fig1.tif

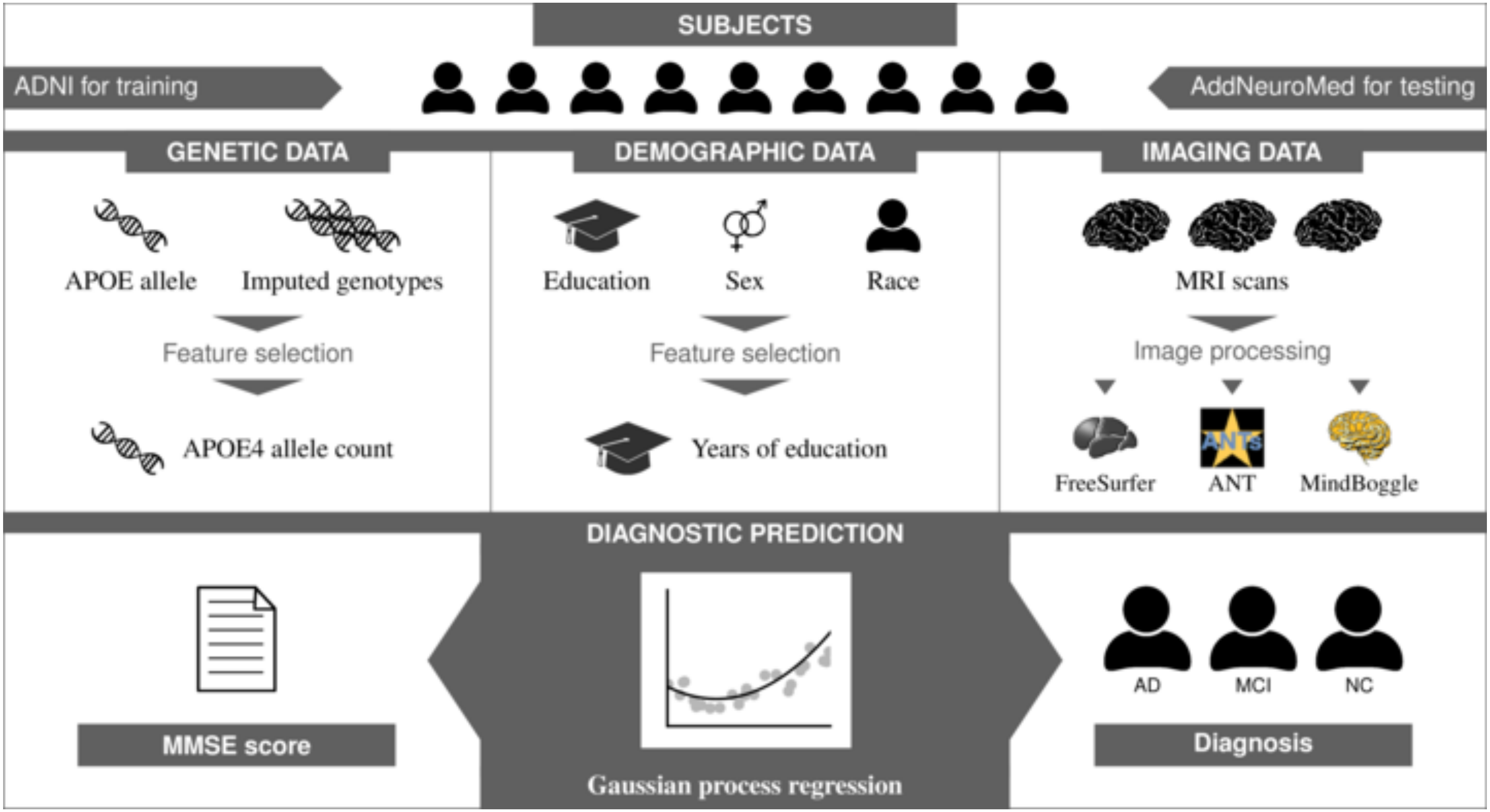

Figure 2

Click here to access/download;Figure;2.tif

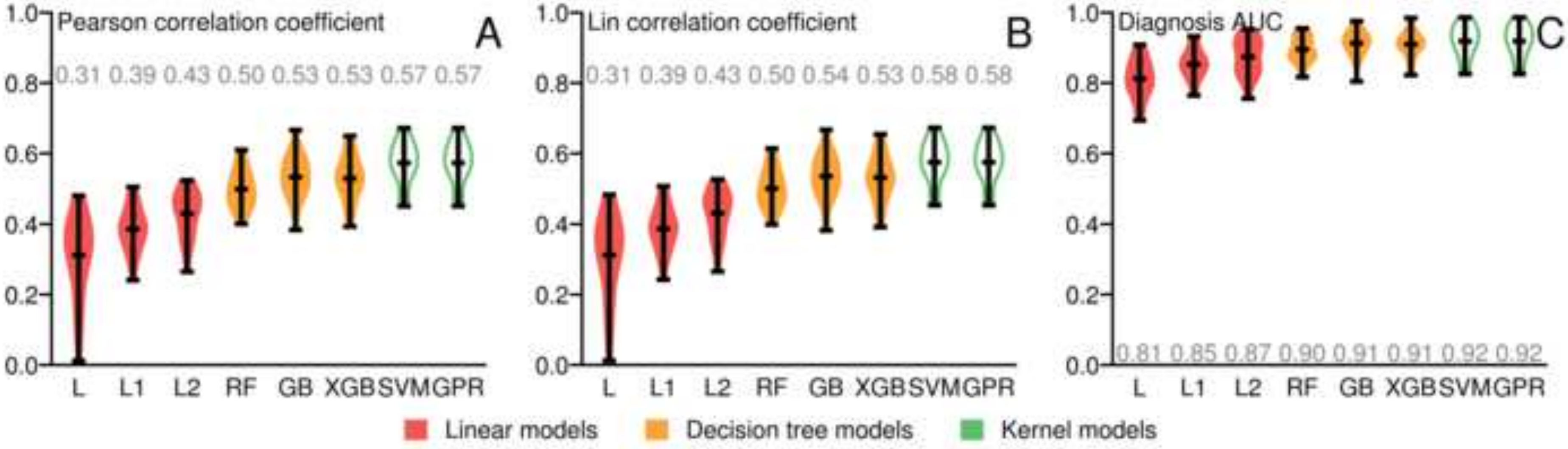

Figure 3

Click here to access/download;Figure;3.tif

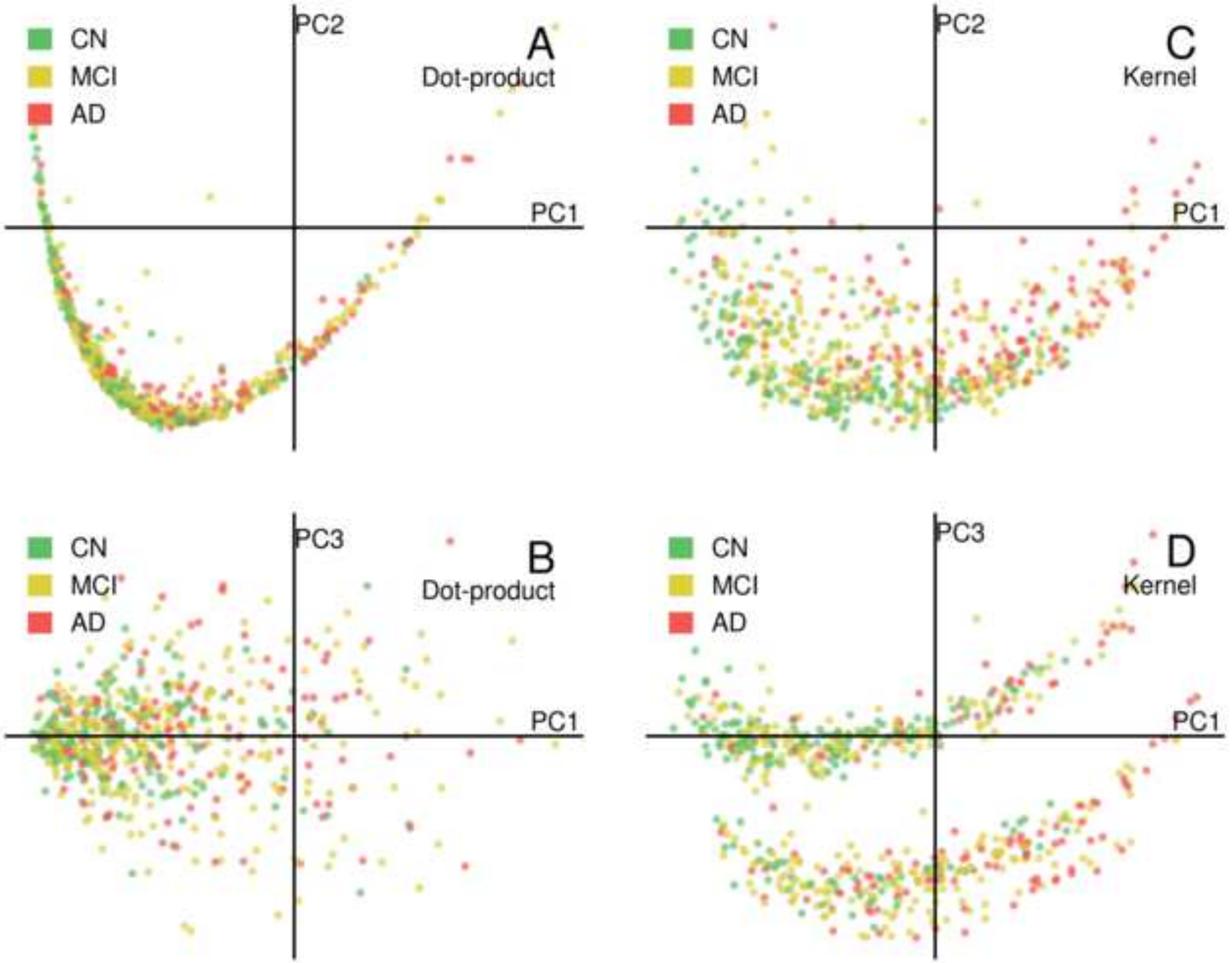

Figure 4

Click here to access/download;Figure;Fig4.tif

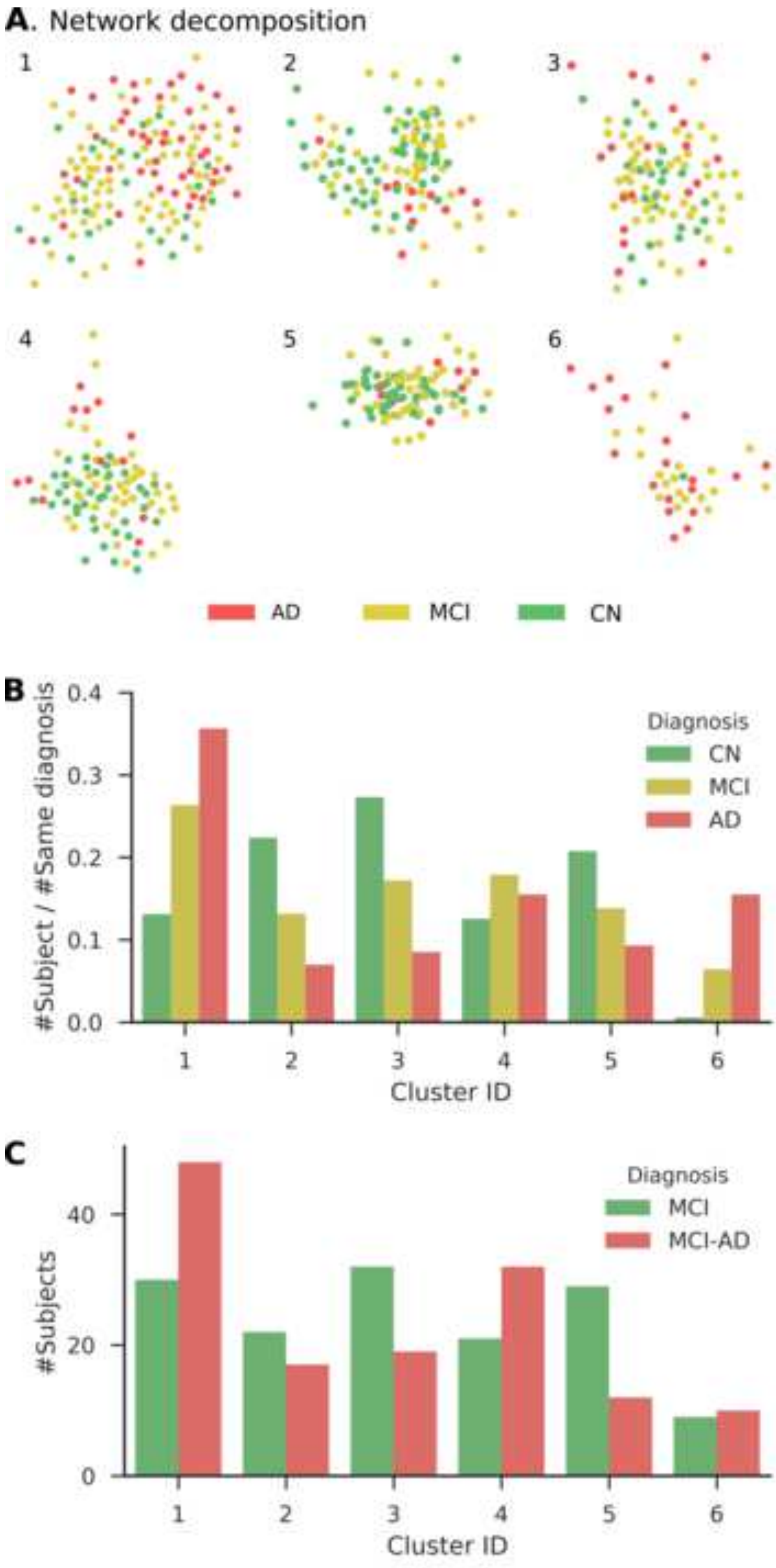

Figure 5

Click here to access/download;Figure;5.tiff

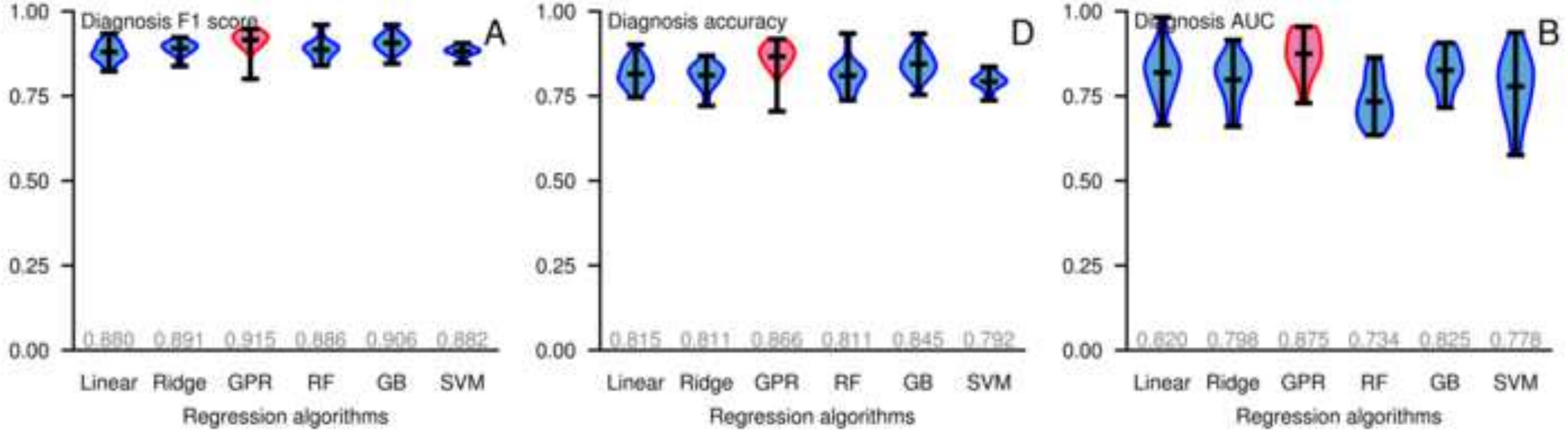

Click here to access/download
Supplementary Material
supplementary.pdf
