# Supplementary material for: A similarity-based approach to leverage multi-cohort medical data on the diagnosis and prognosis of Alzheimer's disease
Source: Gigascience. 2018 Jul 11;7(7):giy085. doi: 10.1093/gigascience/giy085 (PMC6054197; doi:10.1093/gigascience/giy085)
Supplement: Supplemental Files [file giy085_supplemental_files.pdf]

A similarity-based approach to leverage multi-cohort medical data on the diagnosis and prognosis of Alzheimer's disease

Materials and methods

Datasets

The Alzheimer's disease datasets were provided by the Alzheimer's Disease Big Data DREAM Challenge. There were three datasets involved in this study: A training dataset of 628 individuals from ADNI Phase 1 study (Mueller et al. 2005; Jack et al. 2008), a leaderboard validation dataset of 94 individuals from AddNeuroMed study (Lovestone et al. 2009), and a final validation dataset of 88 individuals from AddNeuroMed study. For each individual, a panel of demographic information, APOE $\varepsilon 4$ allele information, and a 1.5T MRI scan was collected and provided to the participants of the challenge. MMSE scores and final clinical diagnoses of individuals in the training dataset were made available to the participants, too. Those of subjects in the two validation datasets were withheld throughout the challenge. A demographic summarization of all three datasets is shown in **Supplementary Table 2&3**. The MRI scans were preprocessed by FreeSurfer (Fischl 2012), ANT (Tustison et al. 2014), and MindBoggle (Klein and Hirsch 2005) to extract numeric features such as surface areas, volumes, cortical thicknesses of brain anatomical structures. See more information about the challenge settings at https://www.synapse.org/#!Synapse:syn2290704.

The training dataset was obtained from the Alzheimer's Disease Neuroimaging Initiative (ADNI) database (adni.loni.usc.edu). The ADNI was launched in 2003 as a public-private partnership, led by Principal Investigator Michael W. Weiner, MD. The primary goal of ADNI has been to test whether serial magnetic resonance imaging (MRI), positron emission tomography (PET), other biological markers, and clinical and neuropsychological assessment can be combined to measure the progression of mild cognitive impairment (MCI) and early Alzheimer's disease (AD).

The Parkinson's disease datasets were collected from the Parkinson's Progression Markers Initiative (The Parkinson's Progression Markers Initiative 2011). The dataset consists of 302 individuals (239 patients and 63 controls, SWEDD excluded). For each individual, a panel of demographic information and a 1.5T MRI scan was collected. The MRI scans were preprocessed by FreeSurfer (Fischl 2012) to extract numeric features such as surface areas and volumes of brain anatomical structures.

Diagnostic model evaluation

We evaluated our models using multiple metrics. The performance of cognitive impairment estimation is measured in both Pearson correlation coefficient and Lin's concordance correlation coefficient between the measured and predicted MMSE scores. They are defined as:

$$\rho_{\text{Pearson}} = \frac{1}{n-1} \sum_{j=1}^{n} \left(\frac{x_j - \bar{x}}{\sigma_x} \right) \left(\frac{y_j - \bar{y}}{\sigma_y} \right)$$

$$\rho_{\text{Lin}} = \frac{2\rho_{\text{Pearson}}\sigma_x\sigma_y}{\sigma_x^2 + \sigma_y^2 + (\bar{x} - \bar{y})^2}$$

where n is the total number of prediction targets, x_j and y_j are respectively clinical and predicted MMSE scores of the j-th subject, $\bar{x}$ and σ_x are respectively the mean and the sample standard deviation of $x_1, \ldots, x_n$, and $\bar{y}$ and σ_y are respectively the mean and the sample standard deviation of $y_1, \ldots, y_n$.

The performance of diagnosis is measured in terms of AD/MCI diganosis F1-score and area under receiver operating characteristic curve (AUC). F1-score is defined as:

$$F_1 = \frac{2 \cdot \text{precision} \cdot \text{recall}}{\text{precision} + \text{recall}}$$

$$\text{recall} = \frac{\text{true positive}}{\text{true positive} + \text{false negative}}$$

$$\text{precision} = \frac{\text{true positive}}{\text{true positive} + \text{false positive}}$$

The receiver operating characteristic curve is created by plotting the true positive ratio (the ratio of correctly identified patients out of all AD/MCI subjects) against false positive ratio (the ratio of incorrectly predicted "patients" out of all normal subjects) at various threshold settings. AUC is defined as an integration of the curve from 0 to 1.

Prediction models

When repeatedly trained on the exact same dataset, machine learning algorithms might capture properties that are specific to the training set but no other datasets. This would cause the model to be overfitted to the training data and undermine the performance and robustness of the model on new prediction targets. In order to prevent overfitting during the training, our models are tested through 5-round 5-fold cross-validation (5x5CV) (Kim 2009). During each round of cross-validation, the training dataset is divided into 5 parts randomly. After withholding each part iteratively, the regression model is then trained with the remaining 4 parts and predicted on the withheld data for evaluation.

GPR is a kernel interpolation method. It makes a prediction for the target according to known values "near" the target in the kernel-mapped feature space. Gaussian process regression requires a kernel function to describe the similarity of two sample points. Here we use a squared-exponential kernel function:

$$k\left(x, x'\right)=\sum_{i=1}^{n} \mathrm{e}^{-\frac{\left(x_i-x_i'\right)^2}{2l^2}}$$

where x and x' are two sample points of n dimensions, x_i is i-th feature of x, and l is the bandwidth parameter. Given the training data feature matrix X and target vector Y, the prediction target y' of the testing data point x' can be estimated with:

$$\hat{y}'=k'\left(K+\alpha I\right)Y$$

where α is the noise parameter, I is the identity matrix, and:

$$k'=\begin{bmatrix}k\left(x', X_1\right) & \ldots & k\left(x', X_n\right)\end{bmatrix}^T$$

$$K=\begin{bmatrix}k\left(X_1, X_1\right) & \ldots & k\left(X_n, X_1\right)\\ \vdots & \ddots & \vdots\\ k\left(X_1, X_n\right) & \ldots & k\left(X_n, X_n\right)\end{bmatrix}$$

The diagnosis prediction is a typical classification problem. Considering the nature of machine learning classifiers, which is to predict a continuous score and then classify them into bins, we can use the previous regression model for scoring. In this case, we use 5x5CV to choose thresholds for the three categories.

Network analysis

We constructed a subject-level similarity network. The undirected network connects all training subjects with edges, and the weights of the edges are calculated by the kernel function k. The network contains no self-connecting edges. We then trimmed the network to keep only highest weighted edges. In order to choose the optimal filter threshold, we tested keeping top 0.5%, 1%, 2.5%, 5%, and 10% weighted edges. For each threshold, we applied the community clustering and evaluated their modularity in terms of modularity Q scores. At the same time, we generated 100 random networks for comparison by randomly shuffling edge weights in the original network, trimmed and clustered these networks using all tested thresholds, and calculated their modularity scores. We then determined the significance for original Q scores at each threshold level by counting how many times the randomized Q scores are larger than the original scores. We chose the most significant threshold level that has least number of higher Q scores from clustering random networks. We then apply Girvan-Newman community clustering algorithm on the trimmed network, performed by GLay clustering in clusterMaker2, a Cytoscape plugin.

Supplementary tables

Table 1. The confusion matrix of the Gaussian process diagnostic model's prediction on ADNI training dataset

-	Predict CN	Predict MCI	Predict AD
Diagnosis CN	143	36	4
Diagnosis MCI	83	111	102
Diagnosis AD	11	21	97

Table 2. A summary of the ADNI training dataset provided by the competition.

Entry	AD	MCI	CN	Overall
Sample size	129	296	183	608
Average age	74.8	75.0	75.7	75.2
Average years of education	14.7	15.7	16.1	15.6
% Female	48.8	34.5	47.0	41.3
0 copy of APOE4 allele	42	137	132	311
1 copy of APOE4 allele	60	123	46	229
2 copies of APOE4 allele	27	36	5	68

Table 3. A summary of the AddNeuroMed testing datasets provided by the competition.

Entry	Leaderboard	Final
Sample size	94	88
Average age	73.7	73.8
Average years of education	9.7	9.3
% Female	52.1	51.1
0 copy of APOE4 allele	56	52
1 copy of APOE4 allele	34	28
2 copies of APOE4 allele	4	8

Supplementary figures

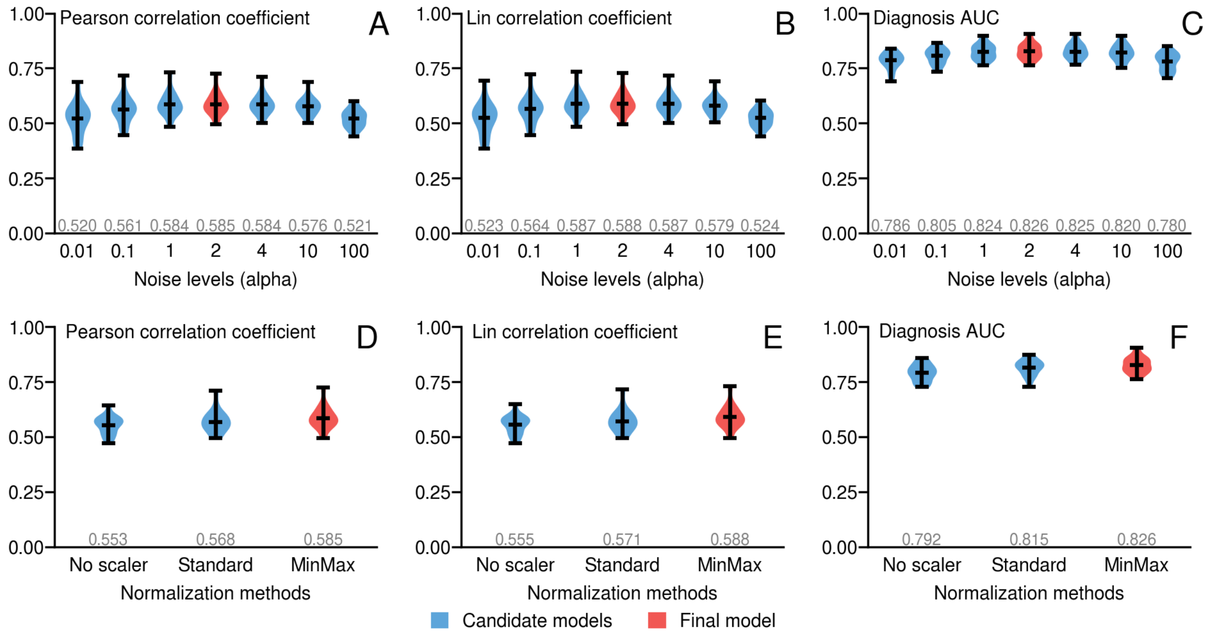

Figure 1: Violin plots of performance of different models estimated by cross-validation. Performance of MMSE regression is evaluated in terms of Pearson product-moment correlation coefficient (A and D) and Lin's concordance correlation coefficient (B and E). Performance of diagnostic predictions is evaluated in terms of AUC (C and F). The average scores are labelled correspondingly. The models chosen for the final submissions are marked with red body. A, B, & C. The performance of different noise parameters α in GPR. D, E, & F. The performance of different preprocessors for MRI data (tested with GPR regressors).

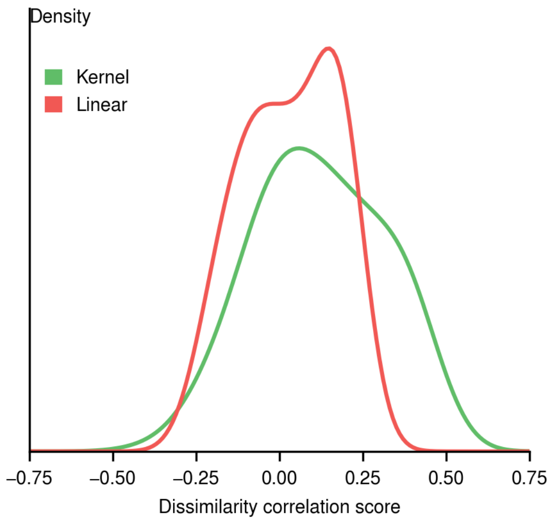

Figure 2: Kernel density estimation of the distribution of dissimilarity correlation score (DCS) of both linear and kernel similarity models (the higher DCS the better).

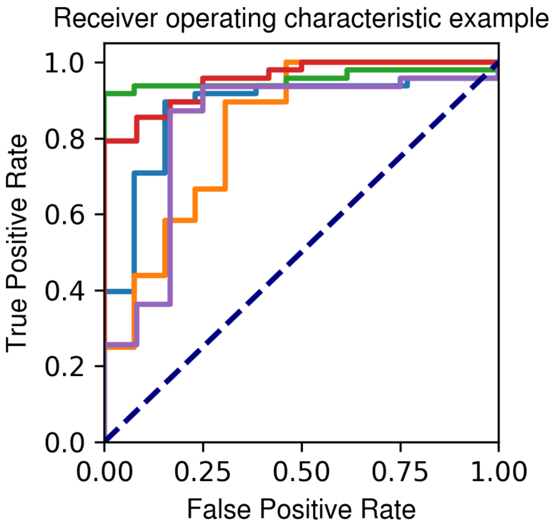

Figure 3: Receiver operating characteristic curve of 5-fold cross validation on Parkinson's disease dataset.
