# Supplementary material for: A similarity-based approach to leverage multi-cohort medical data on the diagnosis and prognosis of Alzheimer's disease
Source: Gigascience. 2018 Jul 11;7(7):giy085. doi: 10.1093/gigascience/giy085 (PMC6054197; doi:10.1093/gigascience/giy085)
Supplement: Response_to_Reviewer_Comments_Original_Submission.pdf [file giy085_response_to_reviewer_comments_original_submission.pdf]

Author's Response To Reviewer Comments

Close

Dear Dr. Scott Edmunds,

We write to submit our revision of the manuscript titled “A similarity-based approach to leverage multi-cohort medical data on the diagnosis and prognosis of Alzheimer's disease”. We have addressed all of the reviewers’ questions in the manuscript. The responses and corresponding changes are attached at the end of the email.

We uploaded to Github all the code associated with the new experiments suggested by reviewers, as well as annotated IPython notebooks of all experiments in the manuscript. The data availability has been changed accordingly. We ensure that readers can reproduce all our results with the data mentioned in the “data availability” section.

We appreciate all reviewer’s constructive suggestions. Thank you for your interest in our work.

Yours sincerely,

Yuanfang Guan
Department of Computational Medicine and Bioinformatics, Palmer Commons
100 Washtenaw Avenue, Ann Arbor, MI 48109-2218
University of Michigan

===

Reviewer #1:

1) Authors used SVM as a regressor candidate algorithm.
Is SVM or SVR ?

Thanks for pointing this out. The regression algorithm is SVR. We have changed all mentioned SVM to SVR.

2) In the examination of the distribution of MCI-to-AD converted patients using similarity network analysis, they constructed a network based on their kernel approach estimated between every pair of subjects with the adopted features.
Then, they applied a density threshold by keeping the 5% of the strongest connections and finally GN algorithm is applied.

It is better to optimize the theshold scheme based on the p-values of the original Q quality values of the clustering algorithm and the one derived by randomize the original similarity matrix by e.g. 1000 times.
BCT contains algorithms for the randomization of a matrix be keeping the strength and the degree of each node.

Thanks for your suggestions. We followed your suggestion. The result is described in Page 8:

“The uneven distribution” (of misclassification cases), “provided that our algorithm predicts diagnosis based on patient similarity, suggests the correlation between the similarity of these MCI patients to AD and normal subjects and their disease progression. More specifically, MCI patients whose conditions resemble those of AD patients might face higher risks of AD conversion, while other MCI patients might be less likely to develop AD. To test the hypothesis, we analyzed the patient similarity network. We built a network that connects subjects in the training dataset with edges. The weights of the edges are the similarity between connecting subjects calculated by the kernel function in our algorithm. Under our hypothesis, highly weighted edges would associate MCI patients that converted later to AD patients more closely than normal subjects in the network. To analyze the connective patterns of highly weighted edges, we first trimmed the network by filtering out the lowest 97.5% weighted edges and then applied Girvan-Newman community clustering algorithm (as implemented in clusterMaker2, a Cytoscape plugin). We chose the threshold by comparing the modularity of the final clustering results against that of trimmed and clustered random networks, and the threshold level of 97.5% achieved the most significant difference (Supplementary Table 4). Girvan-Newman clustering algorithm decomposed the trimmed network into 10 clusters (Fig 4). We dropped the smallest 4 clusters out of the analysis, each of which has less than 10 subjects. Among the remaining clusters, Cluster 1, 3, and 6 contained more normal subjects than AD patients, while Cluster 2, 4, and 5 contains more AD patients. Based on the clustering results, we directly predict that those MCI patients in Cluster 1, 3, and 6 have low risks in disease progression, as well as those in the other clusters have high risks. A Fisher exact test on these subjects confirmed the discriminative power ($p = 0.0001$). Despite not including any longitudinal data, our model successfully captured the properties of the subpopulation which is vulnerable to MCI-to-AD conversion. It demonstrated the effectiveness of the similarity function we adopted in the prediction model.”

The method description is added to the Supplementary Information as a new section:

“We constructed a subject-level similarity network. The network connects all training subjects with edges, and the weights of the edges are calculated by the kernel function k. The network is then trimmed to keep only highest weighted edges. In order to choose the optimal filter threshold, we tested keeping top 0.5%, 1%, 2.5%, 5%, and 10% weighted edges. For each threshold, we applied the community clustering and evaluated their modularity in terms of modularity Q scores. At the same time, we generated 100 random networks for comparison by randomly shuffling edge weights in the original network, trimmed and clustered these networks using all tested thresholds, and calculated their modularity scores. We then determined the significance for original Q scores at each threshold level by counting how many times the randomized Q scores are larger than the original scores. We chose the most significant threshold level that has least number of higher Q scores from clustering random networks. We then apply Girvan-Newman community clustering algorithm on the trimmed network, performed by GLay clustering in clusterMaker2, a Cytoscape plugin.”

3) I would like to see more comments regarding the limitation of the whole process including gaussian distribution and kernel approaches.

Thanks for the question. We appended the following discussion to the last paragraph of the Discussion section (Page 12):

“On the computational side, kernel matrix calculation requires comparing all pairs of samples. The time and memory complexity grows quadratically when the number of samples increases. Researchers have found approximation of kernel calculations for large datasets, but their effects on prediction accuracy of our model needs further investigation.”

===

Reviewer #2:

1) In this paper, for the ADNI data analysis, you choose the APOE4, education levels, and several imaging features. Is there any guideline to choose these covariates?

Thanks for the question. We have extended the method section in regards of feature engineering. We also added a short description on this part in the Results section (Page 3, Line 117-119):

“Hippocampal volumes are chosen because they are the most correlated features and have been described repeatedly in the literature. The remaining features are chosen based on forward feature selection through cross-validation tests.”

2. When you compare your GPR model with other prediction models, could you also try XG-Boost as well? As I know, XG-Boost currently is very popular and of great power in machine learning.

Thank you for the suggestion. We have included the result of XG-Boost into the comparison. The Figure 2 is updated. Given that XG-Boost is similar to general gradient boosting regression tree (except for pruning and binning details), the performance of XG-Boost is very close to gradient boosting regression tree.

Close
