# Supplementary material for: A similarity-based approach to leverage multi-cohort medical data on the diagnosis and prognosis of Alzheimer's disease
Source: Gigascience. 2018 Jul 11;7(7):giy085. doi: 10.1093/gigascience/giy085 (PMC6054197; doi:10.1093/gigascience/giy085)
Supplement: Reviewer_1_Report_(Original_Submission) -- tavros Dimitriadis I Dimitriadis — 12/13/2017 Reviewed [file giy085_reviewer_1_report_(original_submission).pdf]

Reviewer Report

Title: **A similarity-based approach to leverage multi-cohort medical data on the diagnosis and prognosis of Alzheimer's disease**

Version: **Original Submission** **Date:** 12/13/2017

Reviewer name: **tavros Dimitriadis I Dimitriadis**

Reviewer Comments to Author:

Authors demonstrated a novel similarity-based approach that matches individuals to subjects with similar conditions. They modelled the disease with a Gaussian process, and tested the method in the Alzheimer's Disease Big Data DREAM Challenge.

The model predicted patients that are vulnerable to MCI-to-AD conversion through the similarity network.
They also demonstrated the potentiality of the proposed framework.

Authors demonstrated the benefits of kernel-based projection of the features prior to any regression algorithm.

1) Authors used SVM as a regressor candidate algorithm.
Is SVM or SVR ?

2) In the examination of the distribution of MCI-to-AD converted patients using similarity network analysis, they constructed a network based on their kernel approach estimated between every pair of subjects with the adopted features.
Then, they applied a density threshold by keeping the 5% of the strongest connections and finally GN algorithm is applied.

It is better to optimize the theshold scheme based on the p-values of the original Q quality values of the clustering algorithm and the one derived by randomize the original similarity matrix by e.g. 1000 times.
BCT contains algorithms for the randomization of a matrix be keeping the strength and the degree of each node.

3)I would like to see more comments regarding the limitation of the whole process including gaussian distribution and kernel approaches.

Methods

Are the methods appropriate to the aims of the study, are they well described, and are necessary controls included? No

Conclusions

Are the conclusions adequately supported by the data shown? Yes

Reporting Standards

Does the manuscript adhere to the journal's guidelines on minimum standards of reporting? Yes

Choose an item.

Statistics

Are you able to assess all statistics in the manuscript, including the appropriateness of statistical tests used? Yes, and I have assessed the statistics in my report.

Quality of Written English

Please indicate the quality of language in the manuscript: Acceptable

Declaration of Competing Interests

Please complete a declaration of competing interests, considering the following questions:

- Have you in the past five years received reimbursements, fees, funding, or salary from an organisation that may in any way gain or lose financially from the publication of this manuscript, either now or in the future?
- Do you hold any stocks or shares in an organisation that may in any way gain or lose financially from the publication of this manuscript, either now or in the future?
- Do you hold or are you currently applying for any patents relating to the content of the manuscript?
- Have you received reimbursements, fees, funding, or salary from an organization that holds or has applied for patents relating to the content of the manuscript?
- Do you have any other financial competing interests?
- Do you have any non-financial competing interests in relation to this paper?

If you can answer no to all of the above, write 'I declare that I have no competing interests' below. If your reply is yes to any, please give details below.

I declare that I have no competing interests'

I agree to the open peer review policy of the journal. I understand that my name will be included on my report to the authors and, if the manuscript is accepted for publication, my named report including any attachments I upload will be posted on the website along with the authors' responses. I agree for my report to be made available under an Open Access Creative Commons CC-BY license (http://creativecommons.org/licenses/by/4.0/). I understand that any comments which I do not wish to be included in my named report can be included as confidential comments to the editors, which will not be published.

I agree to the open peer review policy of the journal

To further support our reviewers, we have joined with Publons, where you can gain additional credit to further highlight your hard work (see: https://publons.com/journal/530/gigascience). On publication of this paper, your review will be automatically added to Publons, you can then choose whether or not to claim your Publons credit. I understand this statement.

Yes Choose an item.
